# *T*_1/2_ Tuning in a Synergistic BODIPY-Tetrazole Fe(II) Spin Crossover-Photoluminescence System via Counterion Variation

**DOI:** 10.1021/acs.cgd.5c00492

**Published:** 2025-10-13

**Authors:** Martin Huber, Matthias Schöbinger, Berthold Stöger, Michael Reissner, Peter Weinberger

**Affiliations:** † Institute of Applied Synthetic Chemistry, 27259 TU Wien, Getreidemarkt 9, 1060 Vienna, Austria; ‡ X-ray Center, TU Wien, Getreidemarkt 9/164, 1060 Vienna, Austria; § Institute of Solid State Physics, TU Wien, Wiedner Hauptstraße 8, 1040 Vienna, Austria

## Abstract

Multifunctional bistable magnetic materials, particularly spin crossover (SCO)-photoluminescence (PL) systems, are of significant interest for molecular sensor applications. However, achieving predictable SCO tuning while simultaneously combining these properties remains a challenge. In this context, we synthesized a series of heteroleptic Fe­(II) coordination compounds incorporating a fluorescence-active BODIPY-based 1*H*-tetrazole ligand (4,4-difluoro-1,3,5,7-tetramethyl-8-[(1*H*-tetrazol-1-yl)­methyl]-4-bora-3a,4a-diaza-*s*-indacene), with the compounds differing by counteranion size (BF_4_^–^ < ClO_4_^–^ ≪ PF_6_^–^ < CF_3_SO_3_^–^ < SbF_6_^–^). Remarkably, CF_3_SO_3_^–^ coordinates to Fe­(II), while all other anions form noncoordinating isostructural complexes. The coordination of CF_3_SO_3_^–^ can be reversed through a topochemical exchange with H_2_O. Magnetic studies reveal that for the isostructural coordination compounds with noncoordinating anions, increasing anion size leads to incomplete and/or less abrupt spin transitions. The complex incorporating noncoordinating BF_4_^–^ exhibits the most abrupt and complete SCO, demonstrating a weak yet synergistic interplay between SCO and PL signal modulation upon the spin transition, an effect attributed to electronic coupling. While not featuring SCO, the compound with a coordinating CF_3_SO_3_^–^ anion is crystallographically interesting as it features a phase transition, forming a subtle 4-fold superstructure below 180 K. Our study provides a versatile platform for independent tuning of SCO and PL properties, paving the way for application in multifunctional devices.

## Graphical abstract

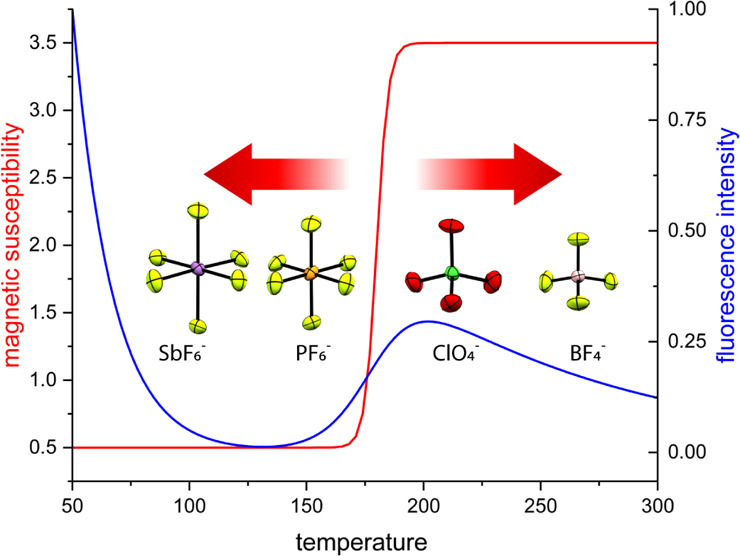

## Introduction

Fe­(II) coordination compounds can undergo reversible switching between a diamagnetic low-spin (LS, *S* = 0) state and a paramagnetic high-spin (HS, *S* = 2) state, a phenomenon known as spin crossover (SCO), which is commonly observed in 3d^4^-3d^7^ metal complexes.^1^ External factors such as thermal, photonic, electrical, and pressure stimuli can induce the spin transition,^2–4^ resulting in notable changes in the structural, optical, magnetic, and vibrational characteristics of the compound.^5–7^

Due to their intrinsic bistability, SCO compounds have significant potential for integration into molecular devices.^8^ In technological applications, a sharp, complete, and hysteretic spin transition at or above room temperature (rt) is highly desirable.^9^

The spin transition can be modulated by three main tuning factors: the counterion, the crystallization solvent (potentially influencing solvate incorporation), and ligand substituents, each of them affecting the electronic and/or packing effects of coordination compounds in the solid state.^10–16^ Electronic effects are primarily controlled by ligand substitution and coordinated anions, while packing effects may be controlled by H-bonding and π–π interactions. These interactions can be mediated by crystal solvents or anions, adding complexity to the predictability of the spin transition. Such intermolecular interactions may enhance cooperativity, resulting in a more abrupt spin transition with hysteresis or, conversely, causing the complex to become trapped in one spin state.^17,18^

Recent advances in SCO chemistry increasingly exploit multifunctionality by coupling the SCO phenomenon with additional physical properties such as photoluminescence (PL),^19–21^ electrical conductivity,^22^ or chiro-optical responses.^23,24^ A synergistic interplay between SCO and the added property paves the way for applications in multifunctional molecular devices. The coupling of SCO with PL is of interest for its utilization in optical sensing^25^ or thermometry,^26,27^ offering an efficient method to reliably read out the spin state by monitoring the luminescence signal.

Over the last 25 years, SCO-PL systems have been developed using a variety of design strategies, which can be generally classified into two main approaches. (I) The single-molecule approach encompasses systems in which the luminophore is incorporated as part of the ligands,^26,28–42^ acts as a counterion in ionic SCO complexes,^43^ or is incorporated into heterodinuclear d-f molecular materials.^44–48^ (II) Nonmolecular composites (grafted systems) combine separate SCO units and fluorophores by doping or decorating the SCO material with fluorophore molecules.^27,49–56^

Single-molecule systems allow for detailed structural characterization, although the synthetic approach is often more intricate and the SCO and/or PL properties are more prone to be altered due to chemical modifications during synthesis. Conversely, nonmolecular composites offer the advantage of independently selecting the SCO and luminophore components, thereby minimizing the risk of significantly altering their individual behavior upon integration.

Regarding the coupling of SCO and PL, the literature differentiates between electronic and structural effects as the primary mechanisms driving SCO-dependent PL modulation. Predominantly, electronic coupling via a spin-state-sensitive energy transfer process has been reported. Efficient electronic coupling requires a substantial spectral overlap between the fluorophore’s emission and the absorption of the SCO center in one of its spin states. The mechanism of energy transfer is strongly influenced by the spatial distance between these components, with a resonant (nonradiative) process preferably occurring at shorter distances, whereas at longer distances, a radiative (emission-reabsorption) process becomes prevalent.^19^ Additionally, structural changes associated with the spin transition can induce mechanical strain effects, thereby further affecting the PL response.^57^

Although achieving a synergistic SCO-PL system remains challenging, an increasing number of coordination compounds incorporating a wide variety of organic fluorophores, including rhodamines,^33^ pyrene,^37,57^ phenazines,^31^ anthracene,^35,43,50,58,59^ and others, have been reported. However, no single candidate has emerged as unambiguously optimal, as each entails a distinct trade-off between the luminescence performance and SCO properties.

Our recent investigations into the photophysical and structural properties of a novel PL-active ligand family have demonstrated its potential for use in a SCO-PL system based on the single-molecule approach.^60^ This fluorescent ligand consists of the well-established fluorophore (4,4-difluoro-4-bora-3a,4a-diaza-*s*-indacene (BODIPY)) and 1*H*-tetrazole (tz) (Chart 1), as the coordinating unit. The BODIPY fluorophore is linked to the *N1* position of 1*H*-tetrazole via a methylene bridge. The benefits of using *N1*-substituted tetrazoles as a coordinating unit for Fe­(II) SCO coordination compounds have been substantiated in previous studies by our group.^15,61–63^

**Chart 1:**
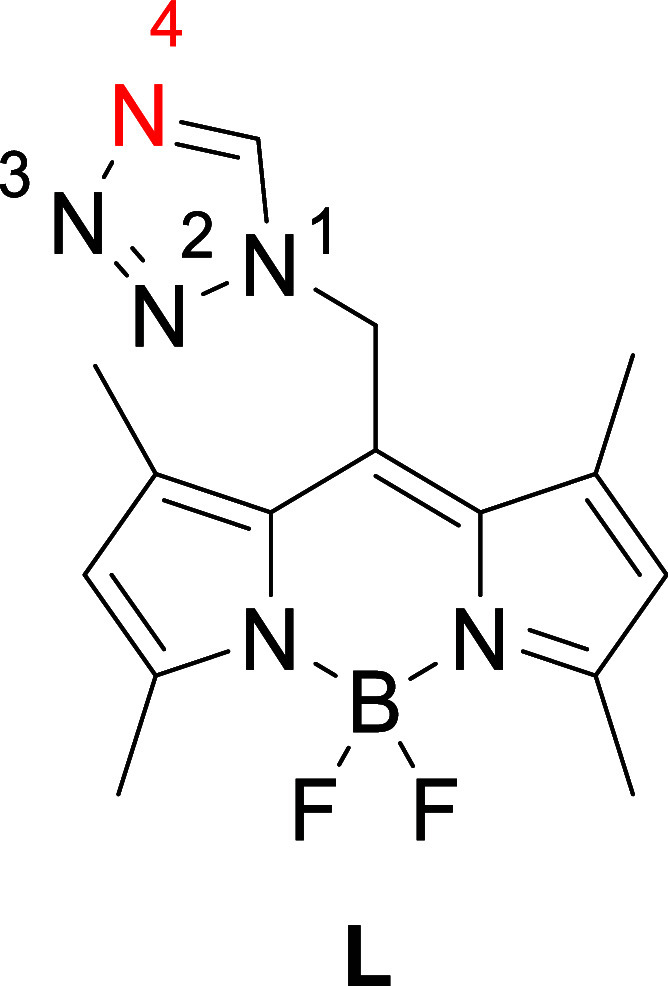
Photoluminescence-Active Ligand **L**, Observed Coordination Site in Red (*N4*-Atom)

The combination of this ligand with different Fe­(II)­X_2_ salts (X = BF_4_^–^, ClO_4_^–^, PF_6_^–^, SbF_6_^–^, and CF_3_SO_3_^–^) enables independent tuning of the SCO and PL properties within a single molecular system, an advantage that is typically limited to grafted systems. By variation of the anion, we aim to achieve a synergistic interplay between SCO and PL, with a sharp spin transition at ambient temperature.

## Results and Discussion

### Synthesis

**L** was synthesized according to an earlier described procedure,^60^ and samples **1**–**6** were synthesized based on protocols for related tz coordination compounds.^64^ The variation of the noncoordinating anions was realized by using different Fe­(II)­X_2_ salts. In the case of **1** (X = ClO_4_^–^) and **2** (X = BF_4_^–^), the corresponding Fe­(II) salt was commercially available. However, for **3** (X = PF_6_^–^) and **4** (X = SbF_6_^–^), the FeX_2_ salt was prepared in situ by an anion exchange through a precipitation reaction of FeBr_2_ and the respective AgX salt.^14^ Subsequently, **L** and the Fe­(II) salt were mixed in acetonitrile (CH_3_CN) and stirred at elevated temperature (40 °C) overnight. After precipitation and washing with diethyl ether (Et_2_O), coordination compounds **1**–**4**, of the general formula [Fe­(**L**)_4_(CH_3_CN)_2_]­(X)_2_·2CH_3_CN, were obtained in moderate yields (Scheme 1).

**Scheme 1:**
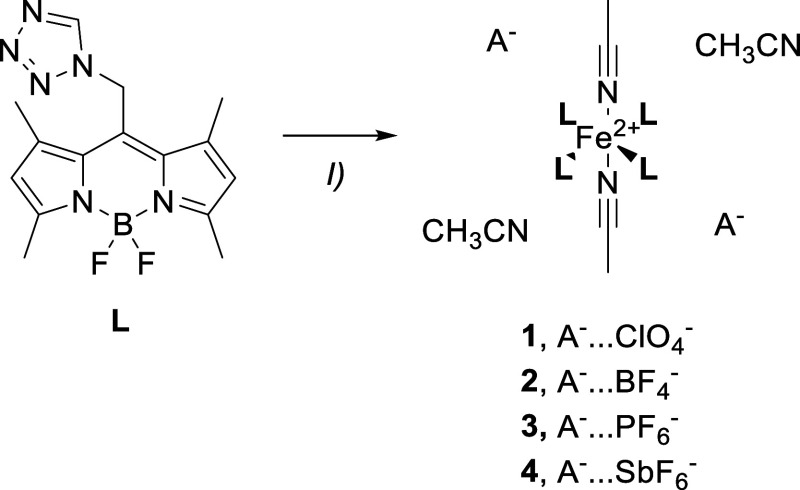
Synthesis of Coordination Compounds **1**–**4**, Reagents and Conditions: (I) Fe­(ClO_4_)_2_·6H_2_O, (**1**) Fe­(BF_4_)_2_·6H_2_O (**2**), Fe­(PF_6_)_2_ (**3**), Fe­(SbF_6_)_2_ (**4**), CH_3_CN, 40 °C, o.n., 50.7% (**1**), 20.7% (**2**), 32.1% (**3**), 25.2% (**4**)

Coordination compound **5a** was prepared in the same manner as that of **1** and **2**, using the corresponding Fe­(II) salt, Fe­(CF_3_SO_3_)_2_, as a direct Fe­(II) source. However, structural analysis (vide infra) revealed that, in addition to **L** and the coligand, the CF_3_SO_3_^–^ anion also coordinates to the metal center, yielding [Fe­(**L**)_2_(CH_3_CN)_2_(CF_3_SO_3_)_2_] (Scheme 2). In **5a**, CF_3_SO_3_^–^ does not act as a noncoordinating anion as originally intended and described in the literature,^65^ disrupting consistency within the series of coordination compounds investigated herein. To achieve the coordination of four molecules of **L** in the equatorial plane, a different strategy was attempted: the use of a less coordinating solvent (acetone instead of CH_3_CN) to prevent solvent coordination. Unfortunately, in the resulting coordination compound **6** [Fe­(**L**)_2_(acetone)_2_(CF_3_SO_3_)_2_], the metal center exhibits a coordination environment similar to that in **5a**, just the coligand was exchanged for acetone (Scheme 2). Due to the fact that the formation of **5a** and **6** required only two equivalents of **L** instead of the four initially used, the excess ligand was removed during workup.

**Scheme 2:**
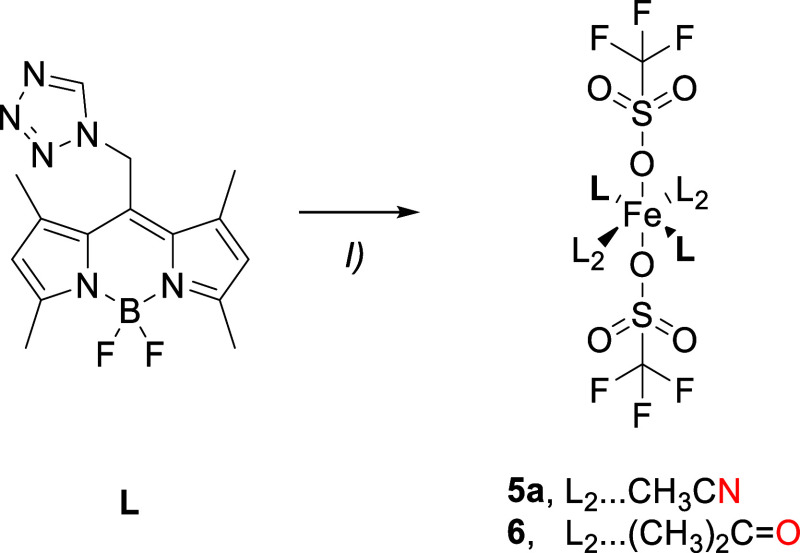
Synthesis of Coordination Compounds **5a** and **6**, Reagents and Conditions: (I) Fe­(CF_3_SO_3_)_2_, CH_3_CN (**5a**), Acetone (6), 40 °C, o.n., 34.9% (**5a**), 45.1% (**6**); Coordination Sites in Red

As a quick indicator of a successful complexation, the shift of the ν_CH(tz)_ band was used. Besides IR spectroscopy, X-ray powder diffraction (XRPD) was deployed to monitor the complexation reactions. Notably, **5b** [Fe­(**L**)_2_(CH_3_CN)_2_(H_2_O)_2_]­(CF_3_SO_3_)_2_ was directly obtained in a single-crystal-to-single-crystal (SC-to-SC) reaction from **5a** (vide infra).

### Crystal Structures

Single crystals (SCs) of coordination compounds **1**–**3**, **5a**, and **6** were obtained via slow diffusion of an antisolvent (Et_2_O) into saturated solutions of CH_3_CN (**1**–**3** and **5a**) or acetone (**6**) at rt. For coordination compound **4**, SCs were grown using the slow evaporation method with CH_3_CN as a solvent under an atmosphere of argon at rt.

Diffraction data of coordination compounds **1**–**4** were collected in the HS state at 300 K and in the LS state at 100 K. However, in the case of **4**, the temperature had to be lowered to the minimal possible measuring temperature of the setup 90 K (Supporting Information), in order to minimize the remaining HS fraction. Compounds **5b** and **6** were measured only at 100 K, as no SCO behavior was expected. Since **5a** exhibits a phase transition, data were collected at 100 and 180 K.

**1**–**4** crystallize in the monoclinic *P*2_1_/*n* space group featuring one crystallographically unique mononuclear complex on a center of inversion. The complex adopts an octahedral geometry and, due to the high steric demand^66^ of **L**, is heteroleptic. While the apical positions are occupied by the coligand (CH_3_CN), the equatorial plane is built by four molecules of **L** (Figure 1), each coordinated via its tetrazolic *N4*-atom (the italicized numbered N-atoms correspond to the tetrazole numbering system in Chart 1). The four **L** molecules consist of two crystallographically independent molecules, which are conformers of **L** in a *cis* arrangement. As revealed by preceding structural and theoretical investigations on **L**, the tz moiety can almost freely rotate around the *N1*–CH_2_ bond.^60^

**Figure 1:**
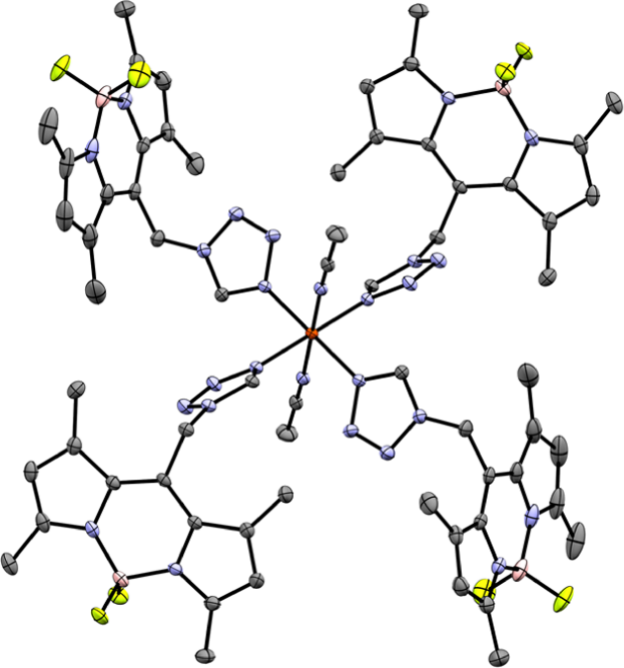
Molecular structure of the mononuclear complex of coordination compound **1** at 100 K (ellipsoids: 50% probability level; atom color code: pink···B, gray···C, blue···N, light green···F, and orange···Fe; and H atoms are omitted for clarity).

For each mononuclear complex in **1**–**4**, there are two solvent molecules (CH_3_CN) and two molecules of the corresponding noncoordinating anion (Scheme 1) located on the general position (Figures S19, S24, S29, and S34). The variation of the anion in **1**–**4** only induces a minimal difference in the spatial arrangement of the ligands (Figures 2, S50–S52) and in the orientation of the tz moiety toward the FBF plane in the conformers of **L** (Table 1). Moreover, the positions of the solvate molecules and the anions are also comparable in the coordination compounds **1**–**4**, thus rendering them isostructural (Table 1, Figures 2, and S50–S52). It has to be mentioned that the structural similarities among coordination compounds featuring anions with equivalent geometry (tetrahedral (**1**–**2**) vs octahedral (**3**–**4**)) are higher than they are between these groups.

**Figure 2:**
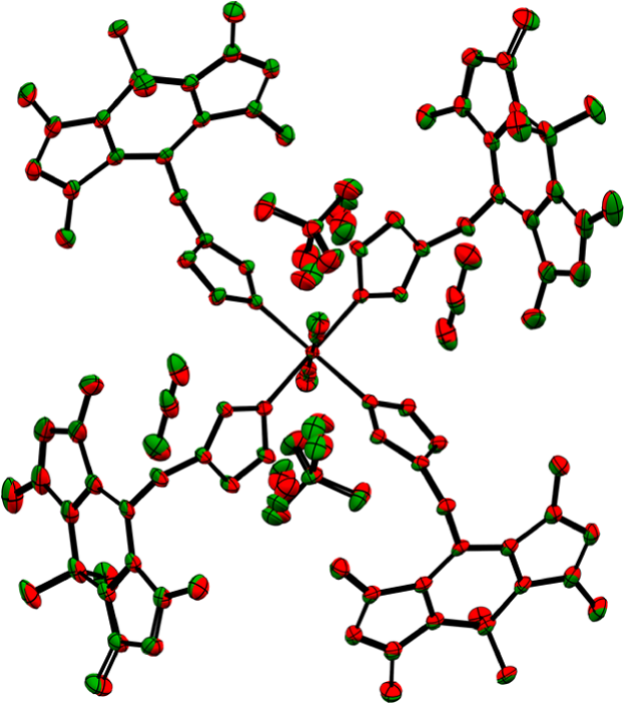
Structure comparison of coordination compound **1** (green) and **2** (red) at 100 K, showing hardly any differences in the spatial arrangement of the ligands, anions, and solvent molecules (ellipsoids: 50% probability level; and H atoms are omitted for clarity).

**Table 1:** Variances of Characteristic Structural Features between Low-Temperature and High-Temperature Measurements of Coordination Compounds **1–4**

compound	temperature /K	spin state	N13(RCN)-Fe1 /Å	N6(tz)-Fe1 /Å^a^	N12(tz)-Fe1 /Å^b^	tz FBF /°^c^	tz FBF /°^d^	Σ^e^ /°	FeNC /°^f^	A^–^-Fe1 /Å^g^	unit cell volume /Å^3^
**1**	100	LS	1.9325(15)	1.9811(13)	1.9735(14)	40.71(16)	37.81(18)	11.8	175.76(14)	5.5432(10)	3939.2(10)
	300	HS	2.121(6)	2.147(5)	2.135(5)	40.7(7)	40.4(8)	18.4	171.8(5)	5.576(2)	4124.6(12)
**2**	100	LS	1.934(2)	1.9833(19)	1.980(2)	41.8(2)	38.6(3)	11.2	175.0(2)	5.464(3)	3902.46(15)
	300	HS	2.130(4)	2.149(3)	2.141(4)	43.0(4)	39.9(5)	17.2	170.5(4)	5.495(7)	4096.29(16)
**3**	100	LS	1.9395(14)	1.9809(13)	1.9795(14)	35.77(16)	37.24(18)	11.8	175.45(13)	5.4414(5)	4033.17(16)
	300	HS	2.151(3)	2.161(2)	2.161(2)	37.3(3)	37.2(3)	21.2	171.0(3)	5.5684(9)	4247.98(16)
**4**	90	LS	1.9516(17)	2.0001(15)	2.00053(16)	34.24(18)	35.9(2)	8.96	175.68(17)	5.6027(4)	4142.8(2)
	300	HS	2.147(4)	2.175(3)	2.171(3)	35.5(5)	36.6(6)	18.4	169.4(5)	5.8046(4)	4365.2(2)

a N1–Fe1 for **1**.

b N7–Fe1 for **1**.

c tz = N1N2N3N4C1, FBF = F1B1F2 for **1** and tz = N3N4N5N6C15, BFF = F1B1F2 for **2**–**4**, underlined atoms face toward the FBF plane.

d tz = N7N8N9N10C16, FBF = F3B2F4 for **1** and tz = N9N10N11N12C30, FBF = F3B2F4 for **2**–**4**, underlined atoms face toward the FBF plane.

e sum of the deviation from 90° of all 12 *cis*-NFeN angles.

f Fe1N13C31 for **1**–**4**.

g Cl1–Fe1 for **1**, B3–Fe1 for **2**, P1–Fe1 for **3**, and Sb1–Fe1 for **4.**

In coordination compounds **1** and **2**, the anions exhibit temperature-dependent disorder. At 100 K, they are disordered about two positions with occupancy ratios of 92.5:7.5(4) for **1** and 95.1:4.9(2) for **2** (Figures S19 and S24), which can be interconverted by rotation along the Cl1–O1 and the B3–B7 axis, respectively. However, the disorder cannot be resolved into distinct positions at 300 K (Figures S20 and S25) because the electron density is too smeared. **3** and **4** show no disorder at both measuring temperatures, which we believe is due to the higher steric demand of the octahedral anions (53.4 Å^3^ for BF_4_^–^ and 54.4 Å^3^ for ClO_4_^–^ vs 73.0 Å^3^ for PF_6_^–^ and 88.7 Å^3^ for SbF_6_^–^).^67^

The spin transition also induces some structural changes, which will be discussed below. However, no major changes in the symmetry or coordination environment are observed for **1**–**4** (Figures S55–S58). For **1**–**3**, the N–Fe bond distances at 100 K (LS) and 300 K (HS) are consistent with the literature for comparable tz coordination compounds.^15,66,68^ However, Fe–N distances in **4** at 90 K suggest an incomplete LS state due to the low *T*_1/2_ (vide infra). The characteristic bond elongation of N–Fe (7.5–9.2%) is accompanied by an increase of the unit cell volume (4.5–5.1%, Table 1). The heteroleptic complex in **1**–**4** is already distorted in the LS state (N­(coligand)-Fe < N­(tz)-Fe, Table 1). In the HS state, this distortion increases, which is in accordance with the literature^37,42,69–71^ and can be quantified by the octahedral distortion parameter (Σ). Σ is defined by the sum of the deviation from 90° of all 12 *cis*-NFeN angles and increases in **1**–**4** from approximately 9.0–11.8° in the LS state to approximately 17.2–21.2° in the HS state (Table 1). Additionally, the spin transition induces a significant decrease in the FeNC angle of the coligands in **1**–**4** from around 175° in the LS state to 170–171° in the HS state.

At 180 K, coordination compound **5a** (Figures 3 and S37–S40) crystallizes like **1**–**4** in the monoclinic *P*2_1_/*n* space group and features one crystallographically unique mononuclear complex on a center of inversion. In contrast to **1**–**4**, in **5a**, only two molecules of **L** coordinate via their tetrazolic N4-atom in a trans arrangement. The other four coordination sites of the octahedral coordination environment are occupied by two molecules of the coligand (CH_3_CN) and two anion molecules (CF_3_SO_3_^–^). The CF_3_SO_3_^–^ molecules coordinate via the O1 atom (Table 2) and are disordered about two positions with an occupancy ratio of 74.40:25.60(15) (Figure S39).

**Figure 3:**
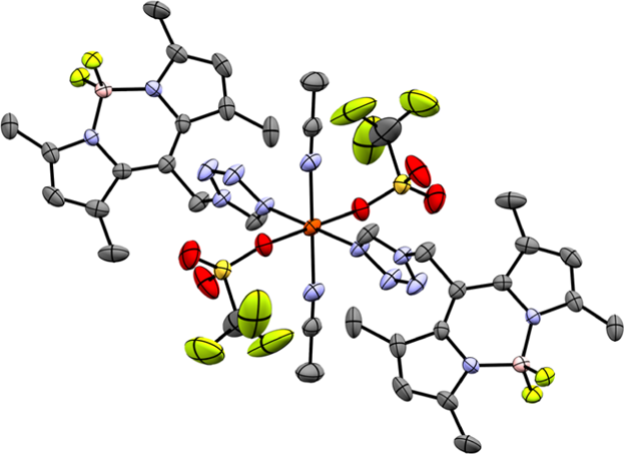
Molecular structure of coordination compound **5a** at 180 K, featuring CH_3_CN as the coligand and CF_3_SO_3_^–^ as the coordinating anion (ellipsoids: 50% probability level; atom color code: pink···B, gray···C, blue···N, light green···F, red···O, yellow···S, and orange···Fe; and H atoms and minor positions of the disordered anion are omitted for clarity).

**Table 2:** Bond Distances and Other Characteristic Structural Features of Coordination Compounds **5**–**6**

compound	*T* /K	spin state	L2-Fe1 /Å^a^	N6(tz)-Fe1 /Å	O1–Fe1 /Å	tz FBF /°^b^	Σ /°	FeL2 /°^c^	A^–^-Fe1 /Å	unit cell volume /Å^3^
**5a**	180	HS	2.1666(12)	2.1601(11)	2.0691(18)	11.64(11)	36.3	173.27(11)	coordinated	2377.2(2)
**5b**	100	HS	2.139(7)	2.194(6)	2.086(5)	17.5(8)	18.8	186.1(7)	^d^	2440.29(19)
**6**	100	HS	2.1311(11)	2.1617(12)	2.1031(11)	45.02(16)	32.2	140.84(11)	coordinated	1198.10(8)

a L2 = CH_3_CN for **5a** (N7–Fe) and **5b** (N7–Fe1) and acetone for **6** (O4–Fe1).

b tz = N3N4N5N6C15, FBF = F1B1F2 for **5a** and **6**; tz = N3N4N5N6C15, FBF = F4B1F5 for **5b**. Underlined atoms face toward the FBF plane.

c Fe1N7C16 for **5a** and **5b**, and Fe1O4C18 for **6**.

d 5.0924­(18) (S1–Fe1) and 5.471(9) (C18–Fe1).

For **5a**, no SCO behavior is observed, and the bond lengths (N–Fe, Table 2) suggest an HS state at the measurement temperature of 180 K.

On cooling to 100 K, **5a** adopts a 4-fold superstructure (modulation wave vector ***q*** = 1/4a* + 1/4b* - 1/4c*), whose basic structure corresponds to the 180 K phase (Figures S59–S60). Since the corresponding lattice is incompatible with the monoclinic lattice system, the space group symmetry is reduced to *P*1̅, corresponding to an overall symmetry reduction by a factor of 8. Owing to the reduction in point symmetry from 2/*m* to 1̅, the crystal at 100 K is a twin of approximately equal volume ratios (51.2:48.8(2)), with the twin law being the lost point operations (2_[010]_ and m_[010]_). Half of the reflections of the two domains overlap practically perfectly (the main reflections and the second order satellites), whereas the remaining (odd order satellites) are distinct (Figure 4). The superstructure reflections were extremely weak; nevertheless, we were able to derive a structural model by applying long exposition times. In the modulated structure, four crystallographically unique complexes are located at the general position. Against our expectations, the disorder of the CF_3_SO_3_^–^-ion in the 180 K phase was not resolved, but only mildly modulated. This remaining disorder is reflected by one-dimensional diffuse scattering in the direction of the modulation wave vector. Owing to weak diffraction data, only the alternative positions of the S atom were refined, the remaining atoms of the CF_3_SO_3_^–^-ions were modeled using large displacement parameters. When independently refining the occupancies of the eight S atoms (two per complex), those of pairs of closely interacting CF_3_SO_3_^–^-ions refined to practically the same values. These occupancies were therefore constrained to the same values. The four resulting occupancies of the major S positions are 92.8(2)%, 90.6(2)%, 90.5(2)%, and 77.7(2)%. Besides this occupational modulation, only very little positional modulation was observed, which explains the weak satellite intensities.

**Figure 4:**
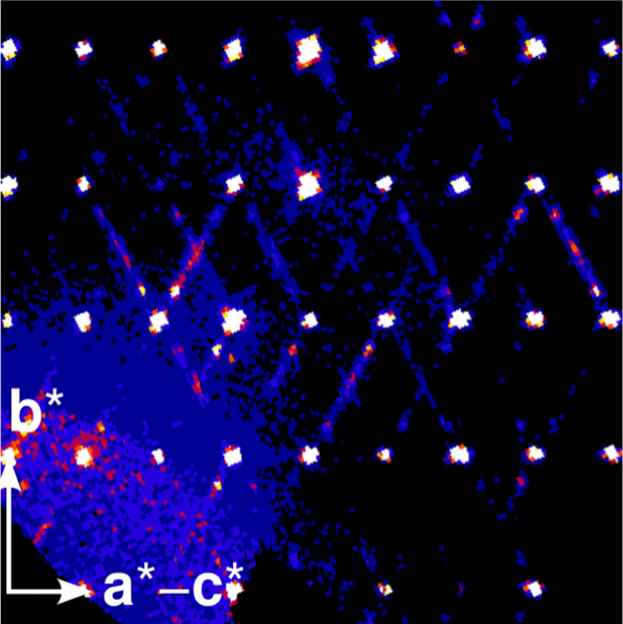
Reciprocal space reconstruction of the (a*-c*,b*) plane passing through 002 (coordinates with respect to the basic structure) of **5a** at 100 K showing extremely weak satellites of both twin domains and streaking in direction of the modulation.

Interestingly, the SCs of **5a** stored under an ambient atmosphere react with air humidity to **5b** (Figures S41–S44) at rt. The water molecules replace the coordinated anion, which subsequently functions as noncoordinating anion. Since, in this SC-to-SC reaction, the symmetry of the educt is retained and the orientation of **5b** is determined by **5a** (Figures S40, S44 and S53), the reaction can be classified as a topochemical reaction, a subgroup of topotactic reactions.^72^ Only small differences in the cell parameters can be reported; however, the cell volume increases from 2377.2(2) Å^3^ to 2440.29(19) Å^3^ due to the incorporation of the water molecules as a ligand. The water molecules are connected by moderate hydrogen bonds^73^ to the CF_3_SO_3_^–^ anions in **5b**, with donor–acceptor distances of 2.779(8) Å (O1–O3) and 2.694(8) Å (O1–O2). Thus, a one-dimensionally periodic hydrogen-bonding network extending in the [010] direction is formed (Figure 5).

**Figure 5:**
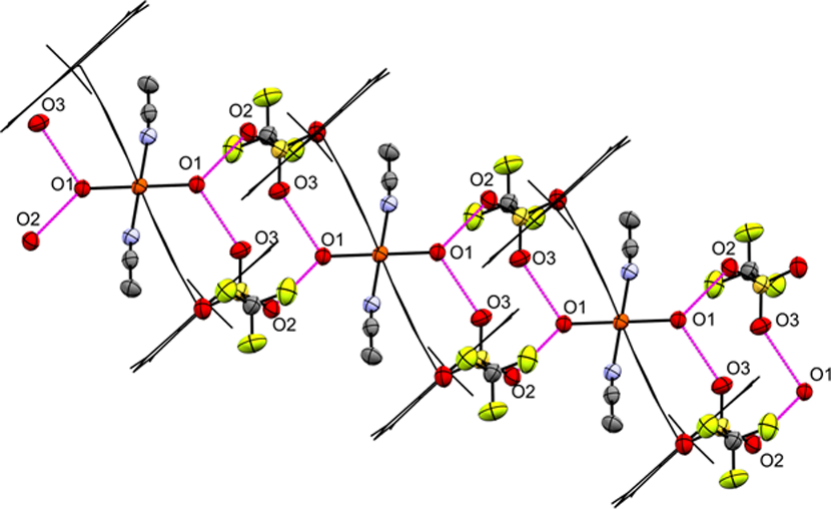
H-bond network in **5b** at 100 K, extending along the crystallographic *b*-axis, with atomic number labeling of donor/acceptor O atoms and H-bonds represented as magenta dotted lines (ellipsoids: 50% probability level; atom color code: gray···C, blue···N, light green···F, red···O, yellow···S, and orange···Fe; and H atoms are omitted for clarity, and **L** molecules are depicted as wireframes for better visualization).

**6** (Figures 6 and S46–S48) crystallizes in the triclinic *P*1̅ space group and features one crystallographically unique mononuclear complex located on a center of inversion. The coordination environment of the Fe­(II) center, except for the coligand (acetone in **6**), is identical to that in **5a**. However, the **L** molecule adopts a different conformation compared to that of **5a** (Table 2). As in **5a**, no SCO behavior is observed, and based on bond lengths, the 100 K measurement suggests that **6** is in the HS state.

**Figure 6:**
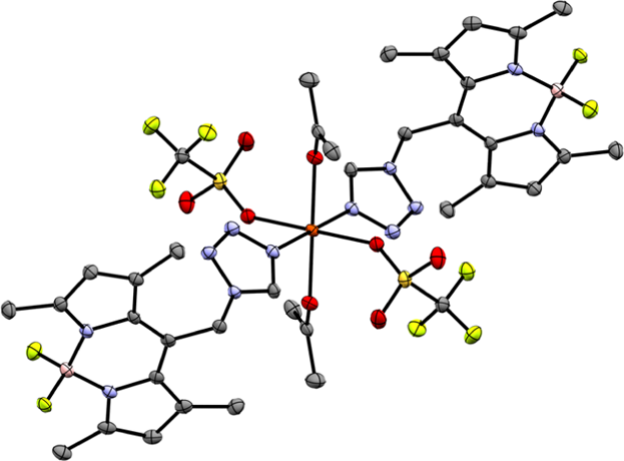
Molecular structure of coordination compound **6** at 100 K, featuring acetone as the coligand and CF_3_SO_3_^–^ as the coordinating anion (ellipsoids: 50% probability level; atom color code: pink···B, gray···C, blue···N, light green···F, red···O, yellow···S, and orange···Fe; and H atoms are omitted for clarity).

According to XRPD, the bulk powders of coordination compounds **1**–**4**, **5a**, and **6** are single phase. For **5b**, no bulk sample was measured since this coordination compound was only synthesized in a SC-to-SC manner.

Magnetic, UV–vis–NIR and PL measurements were performed only for **1**–**4** since for **5a** and **6** no SCO behavior was expected. The analytical data of bulk samples in solid (ATR-IR, UV–vis–NIR, PL, and magnetic susceptibility) discussed in the following sections are shown in the Supporting Information.

### Magnetic Measurements

The magnetic moment was measured at 1 T for powder samples of **1**–**4** in the range from 300 K to 10 K in the cooling mode and subsequently from 10 K to 400 K in the heating mode, followed by a final cooling step from 400 K back to 300 K. The magnetic data are displayed as plots of the molar magnetic susceptibility temperature product χ_M_T versus temperature (Figure 7). The χ_M_T vs T profiles for **1**–**4** in cooling and heating modes are nearly identical, excluding any thermal hysteresis and indicating their thermal stability over the investigated temperature range. Between 350 and 400 K, the coordination compounds **1**–**4** exhibit χ_M_T values of about 3.5 cm^3^ K mol^–1^, which is in line with the expected value for a mononuclear Fe­(II) complex in the HS state (*S* = 2; *g* = 2.2; and χ_M_ *T* = 3.6 cm^3^ K mol^–1^).^74,75^

**Figure 7:**
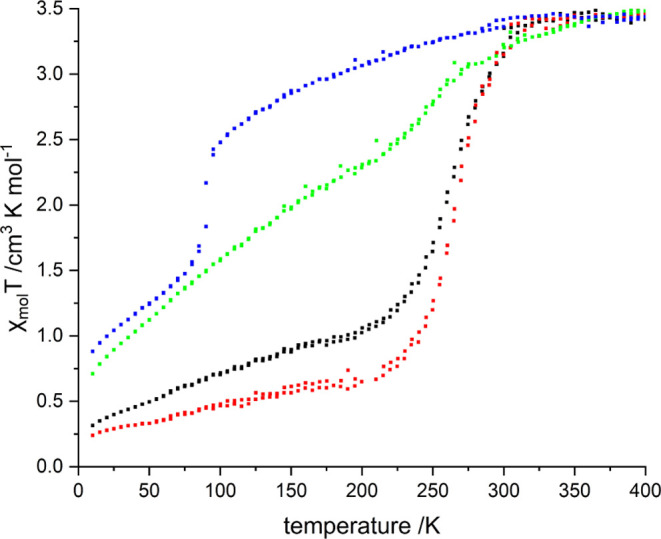
Temperature-dependent magnetic susceptibility measurements for **1** (black), **2** (red), **3** (green), and **4** (blue), shown in the heating mode for clarity.

For compound **1**, χ_M_T decreased gradually from 3.28 cm^3^ K mol^–1^ to 1.30 cm^3^ K mol^–1^ between 305 and 230 K, followed by a further decrease to 0.31 cm^3^ K mol^–1^ until 10 K. This equates to a residual HS fraction of approximately 9% for **1** at 10 K. The spin transition temperature (*T*_1/2_), determined as the maximum of the first derivative of χ_M_T with respect to the temperature (δ­(χ_M_T)/δT), is 265 K.

**2** exhibits a similar, yet more abrupt and complete spin transition; χ_M_T decreases from 3.20 cm^3^ K mol^–1^ to 0.88 cm^3^ K mol^–1^ between 305 and 230 K (*T*_1/2_ = 265 K). Below this temperature, χ_M_T ultimately decreases to 0.24 cm^3^ K mol^–1^, corresponding to a residual HS fraction of 7% at 10 K. Similar findings were reported in a previous study on a molecular Fe­(II) tetrazole system by our group, where a reduction in the number of Fe­(II) centers participating in the spin transition was observed when exchanging the weakly coordinating anion from BF_4_^–^ to ClO_4_^–^.^15^

For coordination compound **3**, χ_M_T first decreases gradually to 3.00 cm^3^ K mol^–1^ until 270 K, then declines more steeply to 2.33 cm^3^ K mol^–1^ until 209 K, followed by a further gradual decrease to 0.71 cm^3^ K mol^–1^ until 10 K, which corresponds to a residual HS fraction of approximately 20%. The *T*_1/2_ for the temperature range with the steepest decrease was determined to be 244 K (δ­(χ_M_T)/δT).

Coordination compound **4**, which incorporates the largest noncoordinating anion, SbF_6_^–^, displays an abrupt yet incomplete spin transition with *T*_1/2_ = 90 K and 26% of Fe­(II) ions remaining in the HS state at 10 K.

All four coordination compounds **1**–**4** are isostructural and exhibit comparable volume changes upon the spin transition of 4.5–5.1%. However, their absolute unit cell volume increases systematically with anion size from BF_4_^–^ to SbF_6_^–^ (vide supra). The observed differences in SCO behavior are therefore primarily attributed to steric and crystal-packing effects of the counterions. Smaller anions (BF_4_^–^ and ClO_4_^–^) occupy the lattice voids of the cationic [Fe­(**L**)_4_(CH_3_CN)_2_]^2+^ framework more efficiently and enforce rigid packing, which promotes stronger elastic interactions between Fe centers. This enhanced lattice cooperativity facilitates abrupt spin transitions at a higher *T*_1/2_. In contrast, larger anions (PF_6_^–^ and SbF_6_^–^) increase the spatial separation between metal centers and reduce elastic coupling, resulting in more gradual or incomplete transitions at lower *T*_1/2_.^76,77^

Another study on the effect of counteranions in Fe­(II) tetrazole systems likewise reported higher spin transition temperatures for the smaller counteranions (BF_4_^–^ and ClO_4_^–^) compared to the larger anions (PF_6_^–^ and SbF_6_^–^).^14^

### Photophysical Measurements

Coordination of **L** to Fe­(II) results in a shift of the ν_CH(tz)_ band from 3133 cm^–1^ to lower wavenumbers in **1** and **6** (both 3129 cm^–1^) and to higher wavenumbers in **2** (3143 cm^–1^), **3** (3147 cm^–1^), **4** (3145 cm^–1^), and **5a** (3144 cm^–1^). In analogous Ag coordination compounds, the same band shifts only to higher wavenumbers as described elsewhere.^64^ In coordination compounds **1**–**5a**, the characteristic ν_CN_ band of the coligand (CH_3_CN) is visible between 2282 cm^–1^ and 2288 cm^–1^.^66^ In **6**, the C=O stretching mode of the coordinated acetone is observable as a very sharp and intense band at 1684 cm^–1^. The aliphatic C–H stretching vibrations (2990 cm^–1^–2870 cm^–1^) of **L** exhibit no significant change due to complexation. Moreover, in the fingerprint region (1600 cm^–1^–400 cm^–1^), the differences of the coordination compounds **1**–**6** are due to additional bands corresponding to each anion. For **1**, the degenerated symmetric bending mode of the ClO_4_^–^ molecule is visible as a band at 621 cm^–1^.^78,79^ The asymmetric deformation mode of the BF_4_^–^ anion is observable at 521 cm^–1^ for compound **2**.^80^ In the case of **3**, two different characteristic bands of the PF_6_^–^ anion are evident. First, the stretching mode appears at 840 cm^–1^ as a broad band, and second, the bending mode appears at 557 cm^–1^ as a sharp band.^81^ In **4**, only the stretching mode of SbF_6_^–^ is visible at 655 cm^–1^ as a broad band.^82^ In coordination compounds **5a** and **6**, the stretching modes of SO and CF appear at 1236 cm^–1^ and 1231 cm^–1^ as well as at 1034 cm^–1^ and 1030 cm^–1^, respectively. The deformation mode of the SO_3_ and the CF_3_ group in **5a**/**6** are visible as bands at 638/634 cm^–1^ and at 515/517 cm^–1^.^83^

Solid-state UV–vis–NIR spectra (Figure S2) indicate that coordination of **L** to Fe­(II) of various Fe­(II)­X_2_ salts does not significantly alter the overall absorption profile. In all spectra, the absorption increases starting below 1000 nm, reaches a maximum around 550 nm, and eventually plateaus with multiple absorption bands, as similarly reported for BODIPY derivatives in the literature.^60,64,84^ As the ^5^T_2_ → ^5^E transition - expected between 800 and 1500 nm for Fe­(II) in the HS state - is typically weak,^15,85^ the strong absorption of BODIPY impedes its identification. Furthermore, this strong absorption obscures the d–d transitions (^1^A_1_ → ^1^T_1_ and ^1^A_1_ → ^1^T_2_), which are characteristic of the Fe­(II) LS state in the 380–800 nm range,^15,39,86^ after cooling, as demonstrated for **2** (Figure S3).

Upon excitation at λ_exc_ = 490 nm, **L** exhibits a broad structureless emission with a maximum (λ_max_) centered at 633 nm.^60^ Coordination to Fe­(II) in coordination compounds **1**, **3**, and **4** causes a slight hypsochromic shift in the PL spectra (λ_exc_ = 405 nm), resulting in λ_max_ shifting to around 615 nm compared to uncoordinated **L**, while the overall spectral profile remains largely unchanged. Notably, coordination compound **4** displays a pronounced shoulder on the low energy side of λ_max_ and an additional sharp peak at 750 nm. In contrast, coordination compound **2** shows a bathochromic shift with λ_max_ at 651 nm and features a pronounced shoulder at 750 nm like in **4 (** Figure S5 **)**.

Emission spectra of the free ligand **L** and coordination compounds **1**–**4** were recorded for solid powder samples over the temperature range from 77 to 298 K in 10 K steps during heating.

For **L**, the highest PL intensity was observed at 77 K (λ_max_ = 649 nm) and a shoulder peak on the higher energy side of the maximum. Upon heating, the PL intensity decreased continuously, the shoulder peak diminished, and λ_max_ shifted hypsochromically to 634 nm at rt (Figures S6, S7). The decrease in the PL intensity with increasing temperature is consistent with thermal quenching, a phenomenon commonly observed in organic fluorophores.

**2** exhibits the highest PL intensity at 77 K (λ_max_ = 645 nm), along with a secondary, weak emission peak at λ_2_ = 747 nm (Figure 8a). As the temperature increases, the overall emission intensity decreases, while the lower-energy peak (λ_2_) becomes more pronounced. Notably, the positions of both emission bands (λ_max_ and λ_2_) remain essentially unchanged throughout the investigated temperature range. From 247 K onward, the decline in emission intensity for both peaks slows down. In particular, the intensity of λ_max_, which initially decreases steeply due to strong thermal quenching, remains almost constant between 247 and 278 K before resuming its downward trend. This atypical thermal behavior coincides with the spin transition temperature of *T*_1/2_ = 265 K as shown in Figure 8b. In contrast, a comparison of **2** with **L, 1, 3**, and **4** does not reveal this behavior, further supporting its dependence on the spin state (Figure S16).

**Figure 8:**
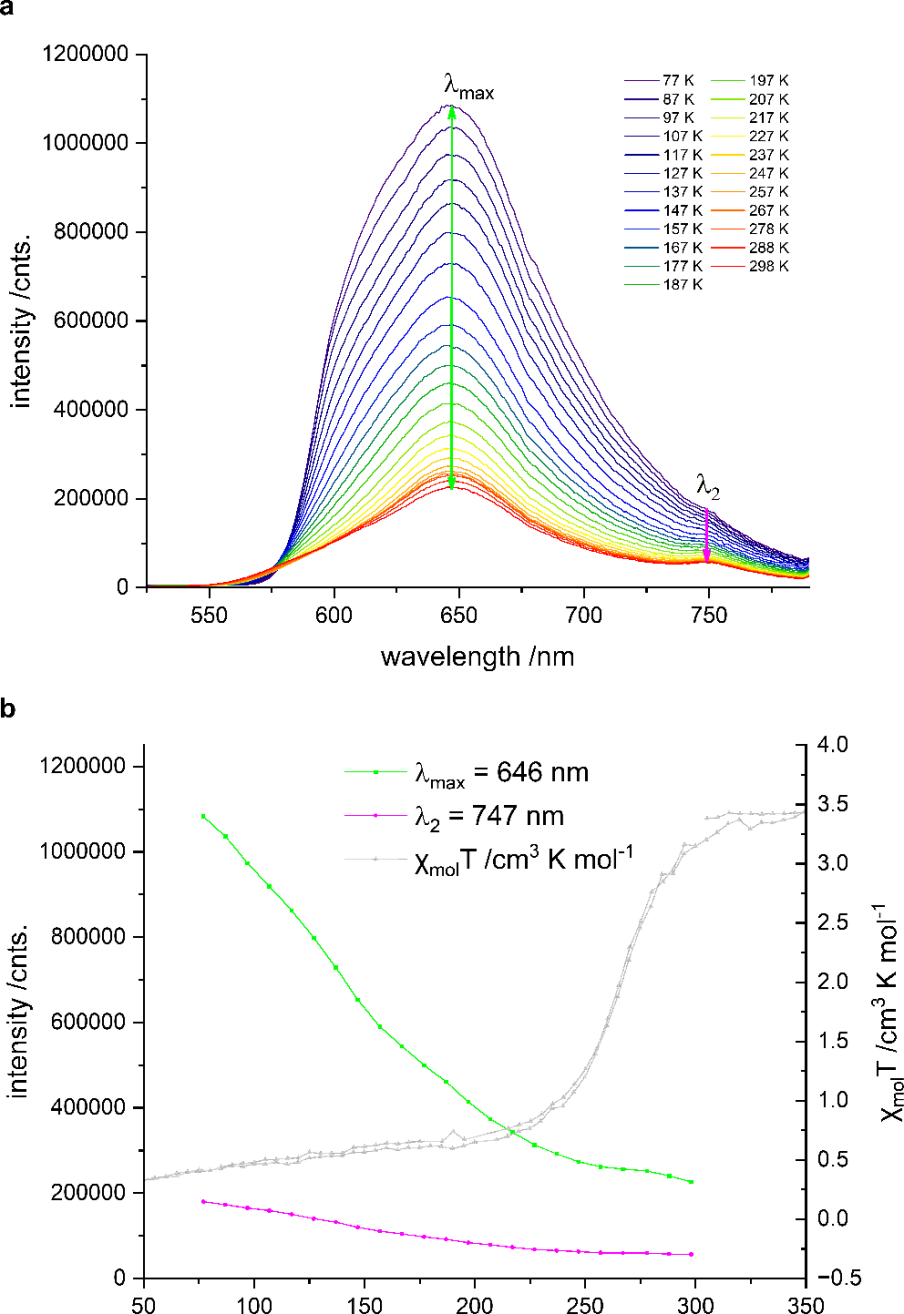
(**a**) Variable temperature PL spectra of **2**. λ_exc_ = 405 nm. Colored arrows indicate the temperature-dependent evolution of the peaks at λ_max_ = 646 nm (green) and λ_2_ = 747 nm (pink). (**b**) Thermal evolution of the PL intensity of the peaks at λ_max_ (green) and λ_2_ (pink). The thermal evolution of χ_M_T of **2** is shown in gray.

For both the ClO_4_^–^ coordination compound **1** and the PF_6_^–^ analogue **3**, the highest emission intensities are also observed at 77 K with λ_max_ = 626 and 646 nm for **1** and **3**, respectively. Neither compound exhibits additional emission features. Upon heating to rt, the PL intensities of both compounds gradually decrease, accompanied by a hypsochromic shift of λ_max_ to 618 nm (**1**) and 617 nm (**3**) (Figures S8, S9 and S12, S13).

In contrast, the SbF_6_^–^ analogue **4** behaves similar to coordination compound **2**, displaying a primary emission peak at λ_max_ = 639 nm and a weaker peak at λ_2_ = 749 nm at 77 K. As the temperature increases, the main maximum shifts hypsochromically to 618 nm, whereas the lower-energy peak remains unchanged. Although the overall PL intensity diminishes significantly upon heating, the reduction in the primary peak is more pronounced than that of λ_2_. Aside from this typical thermal quenching behavior, no additional temperature-dependent anomalies are observed for **4** (Figures S14, S15).

In the four coordination compounds **1**–**4**, strong thermal quenching suppresses PL emission. However, we attribute the slight but clearly visible increase in PL intensity observed in **2** to a synergistic interplay between the intrinsic fluorescence of the BODIPY ligand and the SCO process in Fe­(II). In addition to thermal quenching, we propose that spin-state-dependent PL quenching occurs in the LS state due to a spectral overlap between the emission and the Fe­(II) d–d absorption bands, allowing energy transfer. Upon the spin transition to the HS state (*T*_1/2_ = 265 K), the d–d band of Fe­(II) shifts to the NIR region, resulting in a reduced nonradiative deactivation pathway and thus a relatively enhanced PL intensity.^27,35,50,55,87^

Although there should be a greater spectral overlap in the LS state than in the HS state,^88^ the strong absorption of the BODIPY ligand obscures the d–d transitions of Fe­(II) in both the LS and HS states in the UV–vis spectra (vide supra).

Consequently, both pronounced thermal quenching and strong absorption of the ligand contribute to a weak synergistic effect.

For **1**, **3**, and **4**, which undergo less complete and/or abrupt spin transitions, the modulation effect may be even weaker and, therefore, not detectable. In particular, **4** exhibits an incomplete spin transition at very low temperatures, where the decrease in PL intensity due to thermal quenching is even stronger and dominates the emission behavior.

A detailed structural analysis of compounds **1**–**4** revealed no significant changes in the arrangement of BODIPY units upon the spin transition, ruling out mechanical strain effects as a contributing factor to the synergistic SCO-PL interplay in coordination compound **2** (vide supra). Additionally, structural investigations did not provide an explanation for the temperature-dependent λ_max_ shift observed in **1**, **3** and **4**.

To exclude the possibility that the observed synergy in PL modulation in **2** stems from variations in the absorption at λ_exc_, the temperature-dependent reflectivity (*F*(*R*)) was measured in the heating mode in 10 K steps. *F*(*R*) decreases linearly with increasing temperature, a trend consistent with the literature.^31^ However, this decrease does not correlate with changes in the PL intensity in the spin transition region (Figure S4).

## Conclusion

In this work, we report the successful synthesis of a series of four isostructural heteroleptic Fe­(II) SCO coordination compounds that differ only by their noncoordinating anion (BF_4_^–^, ClO_4_^–^, PF_6_^–^, and SbF_6_^–^). Each Fe­(II) complex features an octahedral coordination environment with four BODIPY luminophores **L** coordinated through a 1*H*-tetrazole moiety and CH_3_CN occupying the apical coordination sites.

Remarkably, the behavior of CF_3_SO_3_^–^ deviates from that of the other employed anions by coordinating directly to Fe­(II) and thereby reducing the number of coordinated ligands of **L** to two. Through a topotactic reaction, an exchange with H_2_O converts CF_3_SO_3_^–^ into a noncoordinating anion.

Magnetic measurements reveal that the SCO characteristics of the isostructural complexes **1**–**4** are modulated by the size of the noncoordinating anion, with larger anions (BF_4_^–^ < ClO_4_^–^ ≪ PF_6_^–^ < SbF_6_^–^) resulting in a less abrupt and/or incomplete spin transitions.

Despite the strong thermal quenching observed in all four complexes, including uncoordinated **L**, complex **2** displays spin-state-dependent PL modulation that overcomes this quenching, correlating with its abrupt and complete SCO behavior. We attribute this synergistic interplay between SCO and PL to electronic coupling effects rather than to any significant structural alterations upon the spin transition.

Overall, the study presents a molecular system in which the SCO and PL properties can be individually tuned. Future efforts will explore the substitution of apical ligands with various nitrile species to achieve a complete and abrupt spin transition at or above rt, which potentially enhances synergistic PL signal modulation.

## Supplementary Material



## Notes

Experimental protocols, crystal structures, and spectroscopic data (UV–vis, PL, and IR spectra). The following files are available free of charge. Supporting Information (PDF) Crystallographic data (CIF) CSD 2440404–2440409 and CSD 2440425–2440431 contain the supplementary crystallographic data for this paper (PDF)
