# Supplementary material for: T 1/2 Tuning in a Synergistic BODIPY-Tetrazole Fe(II) Spin Crossover-Photoluminescence System via Counterion Variation
Source: Cryst Growth Des. 2025 Oct 13;25(21):8875–85. doi: 10.1021/acs.cgd.5c00492 (PMC12593397; doi:10.1021/acs.cgd.5c00492)
Supplement: Supplementary file 1 [file cg5c00492_si_001.pdf]

Supporting Information

$T_{½}$ Tuning in a Synergistic BODIPY-Tetrazole Fe(II) Spin Crossover-Photoluminescence System via Counterion Variation

Martin Huber,*[a]‡ Matthias Schöbinger,[a]‡ Berthold Stöger,[b] Michael Reissner[c] and Peter Weinberger*[a]

[a]

Dr. M. Huber, Dr. M. Schöbinger, Prof. P. Weinberger
Institute of Applied Synthetic Chemistry, TU Wien
Getreidemarkt 9, 1060, Vienna (Austria)
E-mail: martin.e163.huber@tuwien.ac.at, peter.e163.weinberger@tuwien.ac.at

[b]

Dr. B. Stöger
X-ray Center, TU Wien
Getreidemarkt 9/164, 1060, Vienna (Austria)

[c]

Prof. M. Reissner
Institute of Solid State Physics
Wiedner Hauptstraße 8, 1040, Vienna (Austria)

[‡]

These authors contributed equally to this manuscript.

Table of Contents

Experimental protocols ... 3

Magnetic measurements ... 4

Spectroscopic measurements ... 4

UV-Vis-NIR spectra ... 5

PL spectra ... 7

Ligand L ... 8

Coordination compound 1 ... 9

Coordination compound 2 ... 10

Coordination compound 3 ... 11

Coordination compound 4 ... 12

XRPD measurements ... 13

X-Ray Structure Determination ... 17

References ... 40

IR spectra ... 41

Experimental protocols

All syntheses were performed with dried glassware, anhydrous and degassed solvents, and under an atmosphere of argon. Reaction monitoring was performed by IR-spectroscopy and XRPD. The ligand **L** (4,4-difluoro-1,3,5,7-tetramethyl-8- [(1H-tetrazol-1-yl)methyl]-4-bora-3a,4a-diaza-s-indacene) was synthesized according to a literature protocol[1], all other starting materials were commercially purchased and used without further purifications if not stated otherwise.

Synthesis of coordination compounds 1

$Fe(ClO_4)_2{\bullet}6H_2O$ (13.7 mg, 0.0379 mmol, 1.00 equiv.) and a spatula tip of ascorbic acid were mixed in CH_3CN (1 mL), filtrated and added to a mixture of **L** (50.0 mg, 0.151 mmol, 4.00 equiv.) and CH_3CN (2 mL). After the reaction mixture was stirred at 40 °C over night, it was cooled to room temperature and approx. 90 % of the solvent was evaporated, whereupon the formation of a precipitate could be reported. Diethyl ether (8 mL) was added to facilitate the precipitation of the crude product. The crude product was separated from the supernatant, washed with diethyl ether (3 x 6 mL) and dried in high vacuum to yield 31.8 mg (50.7 %) of **1** as dark red solid.

Synthesis of coordination compounds 2

$Fe(BF_4)_2{\bullet}6H_2O$ (12.9 mg, 0.0370 mmol, 1.00 equiv.) and **L** (50.0 mg, 0.151 mmol, 4.00 equiv.) were mixed in CH_3CN (3 mL). After the reaction mixture was stirred at 40 °C for 4 hours, it was cooled to room temperature and approx. 90 % of the solvent was evaporated, whereupon the formation of a precipitate could be reported. Diethyl ether (8 mL) was added to facilitate the precipitation of the crude product. The crude product was separated from the supernatant, washed with diethyl ether (3 x 6 mL) and dried in high vacuum to yield 12.8 mg (20.7 %) of **2** as dark red solid.

Synthesis of coordination compounds 3

$FeBr_2$ (8.19 mg, 0.0380 mmol, 1.00 equiv.) and $AgPF_6$ (19.6 mg, 0.0760 mmol, 2.00 equiv.) were separately dissolved each in CH_3CN (0.5 mL). After combining the solutions, a precipitation formed, which was separated by filtration and the supernatant was added to a mixture of **L** (50.2 mg, 0.152 mmol, 4.00 equiv.) and CH_3CN (2 mL). After the reaction mixture was stirred at 40 °C over night, it was cooled to room temperature and approx. 90 % of the solvent was evaporated, whereupon the formation of a precipitate could be reported. Diethyl ether (8 mL) was added to facilitate the precipitation of the crude product. The crude product was separated from the supernatant, washed with diethyl ether (3 x 6 mL) and dried in high vacuum to yield 21.3 mg (32.1 %) of **3** as dark red solid.

Synthesis of coordination compounds 4

$FeBr_2$ (8.19 mg, 0.0380 mmol, 1.00 equiv.) and $AgSbF_6$ (26.4 mg, 0.0760 mmol, 2.00 equiv.) were separately dissolved each in CH_3CN (0.5 mL). After combining the solutions, a precipitation formed, which was separated by filtration and the supernatant was added to a mixture of **L** (50.2 mg, 0.152 mmol, 4.00 equiv.) and CH_3CN (2 mL). After the reaction mixture was stirred at 40 °C over night, it was cooled to room temperature and approx. 90 % of the solvent was evaporated, whereupon the formation of a precipitate could be reported. Diethyl ether (8 mL) was added to facilitate the precipitation of the crude product. The crude product was separated from the supernatant, washed with diethyl ether (3 x 6 mL) and dried in high vacuum to yield 18.5 mg (25.2 %) of **4** as dark red solid.

Synthesis of coordination compounds 5a and 6

$Fe(CF_3SO_3)_2$ (13.5 mg, 0.0380 mmol, 1.00 equiv.) and **L** (50.2 mg, 0.152 mmol, 4.00 equiv.) were mixed in CH_3CN (3 mL) for **5a** or in acetone (3 mL) for **6**. After the reaction mixture was stirred at 40 °C over night, it was cooled to room temperature and approx. 90 % of the solvent was evaporated, whereupon the formation of a precipitate could be reported. Diethyl ether (8 mL) was added to facilitate the precipitation of the crude product. The crude product was separated from the supernatant, washed with diethyl ether (3 x 6 mL) and dried in high vacuum to yield 23.3 mg (34.9 %) of **5a** or 30.1 mg (45.1 %) of **6** as dark red solid.

Magnetic measurements

Magnetic measurements were performed using a Physical Property Measurement System (Quantum Design). The measurement setup employed a vibrating sample magnetometer (VSM) insert, and brass sample holders with quartz-glass powder containers. The magnetic moment was measured by applying an external field of 1 T, first in cooling mode from 300 K to 10 K, subsequently in heating mode from 10 K to 400 K, and finally in a cooling step from 400 K back to 300 K, with data collection in 5 K steps after thermal stabilization. Corrections for the diamagnetic contribution of the sample holder were applied by subtracting results of blank measurements with $Gd_2(SO_4)_3$.

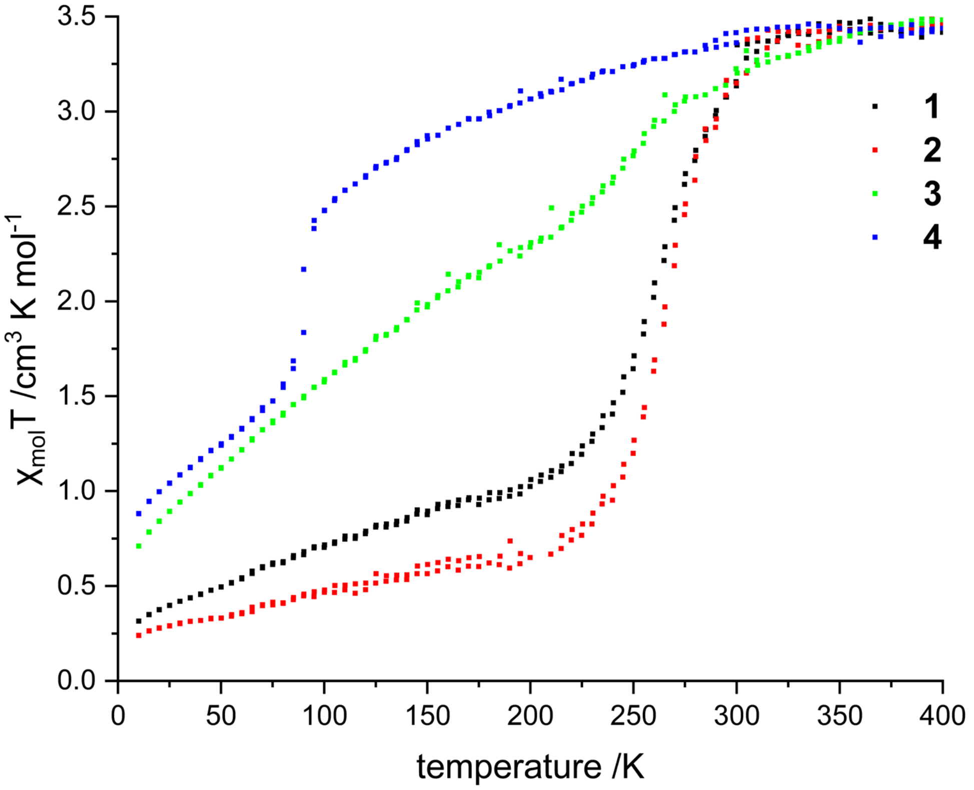

Figure S 1. Temperature-dependent magnetic susceptibility measurements for **1-4**, shown in heating mode.

Spectroscopic measurements

UV-Vis-NIR measurements were performed on a Lambda 900 spectrophotometer (PerkinElmer). Solid samples were measured in diffuse reflectance with a Harrick powder sample holder in “Praying Mantis” configuration against $BaSO_4$. Diffuse reflectance data (R) were converted using the Kubelka-Munk function, defined as $F(R) = \frac{(1-R)^2}{2R}$.

IR spectra were recorded with a SpectrumTwo ATR-FT-IR-spectrometer (Perkin Elmer) using a diamond ATR-unit.

All PL spectra were recorded with a FluoTime 300 fluorescence lifetime spectrometer (PicoQuant) equipped with a coaxial UV-Xenon arc lamp, a high-resolution excitation and emission double monochromator, and a PMA hybrid 07 detector. Variable temperature experiments were performed using a liquid-nitrogen-filled cryostat Optistat DN connected to a MercuryiTC temperature controller (Oxford Instruments).

UV-Vis-NIR spectra

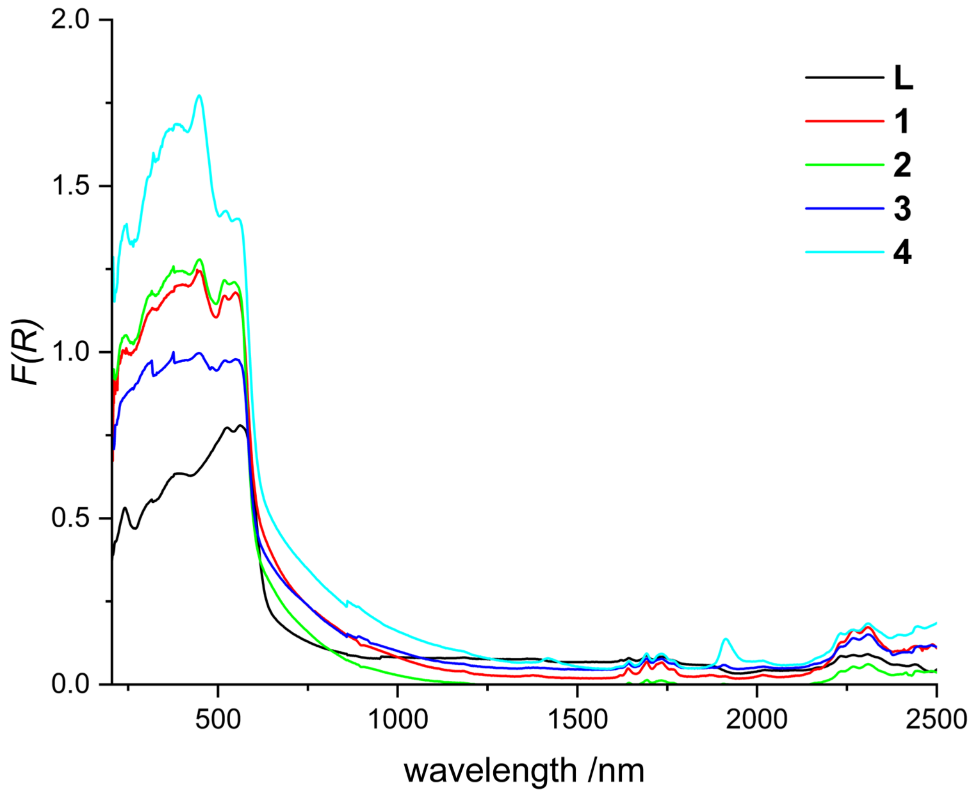

Figure S 2. UV-Vis-NIR spectra of **L** and **1-4**.

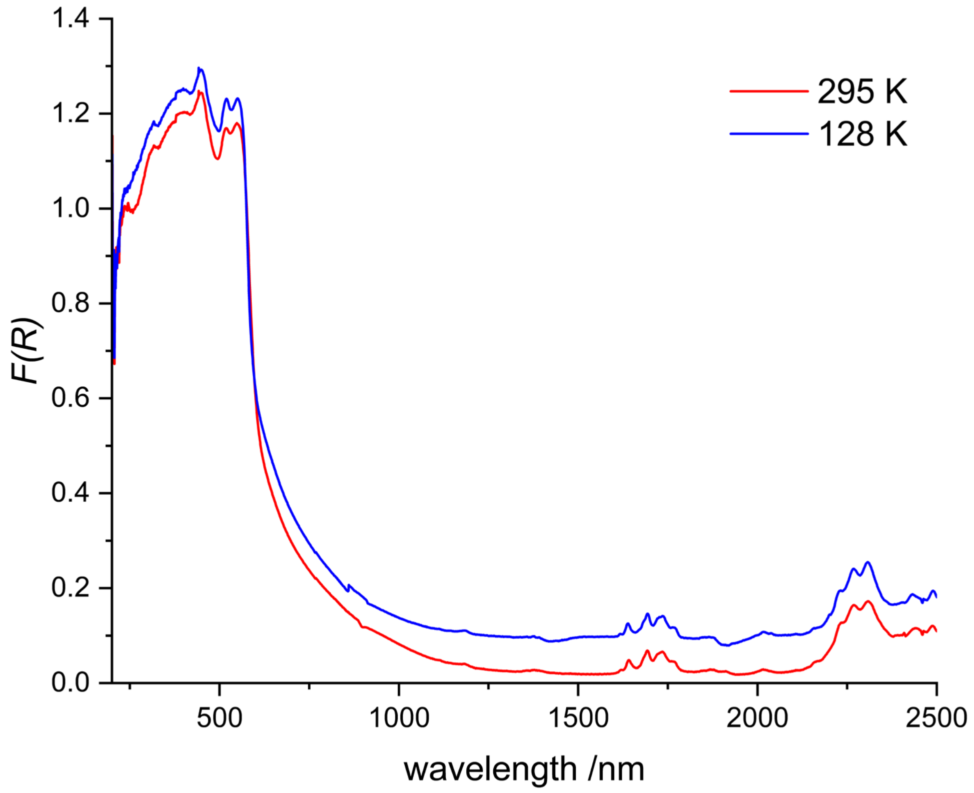

Figure S 3. UV-Vis-NIR spectra of **2** measured at 295 K (red) and 128 K (blue).

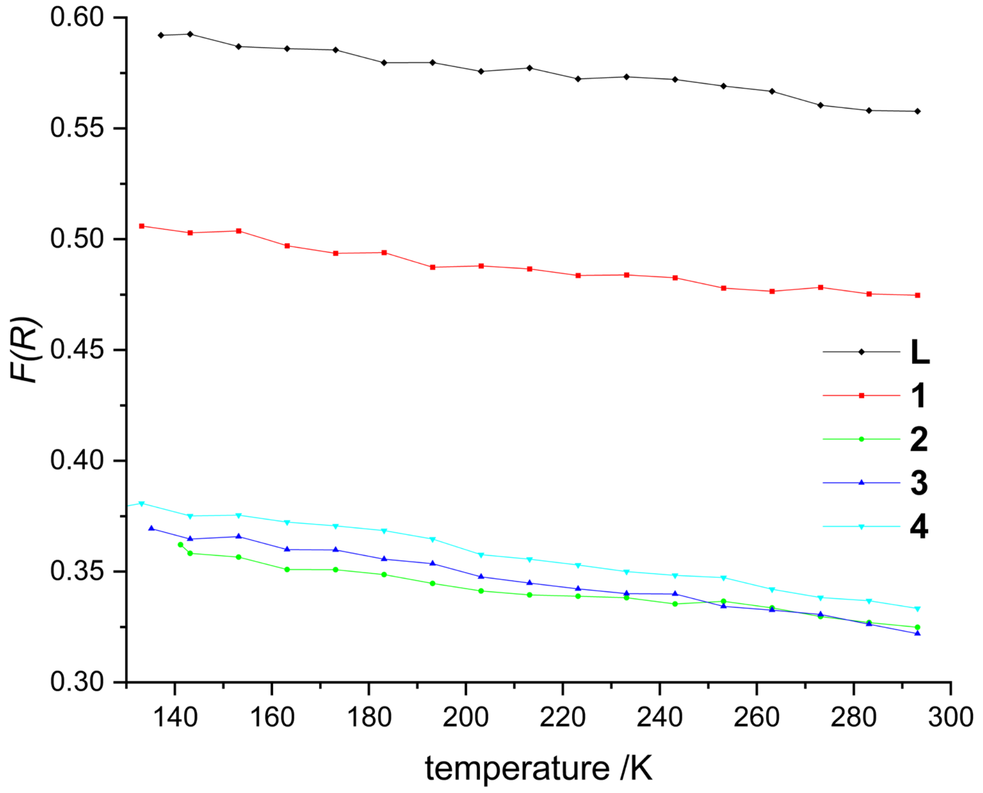

Figure S 4. Temperature dependent reflectivity data for **L** at 490 nm and **1-4** at 405 nm.

PL spectra

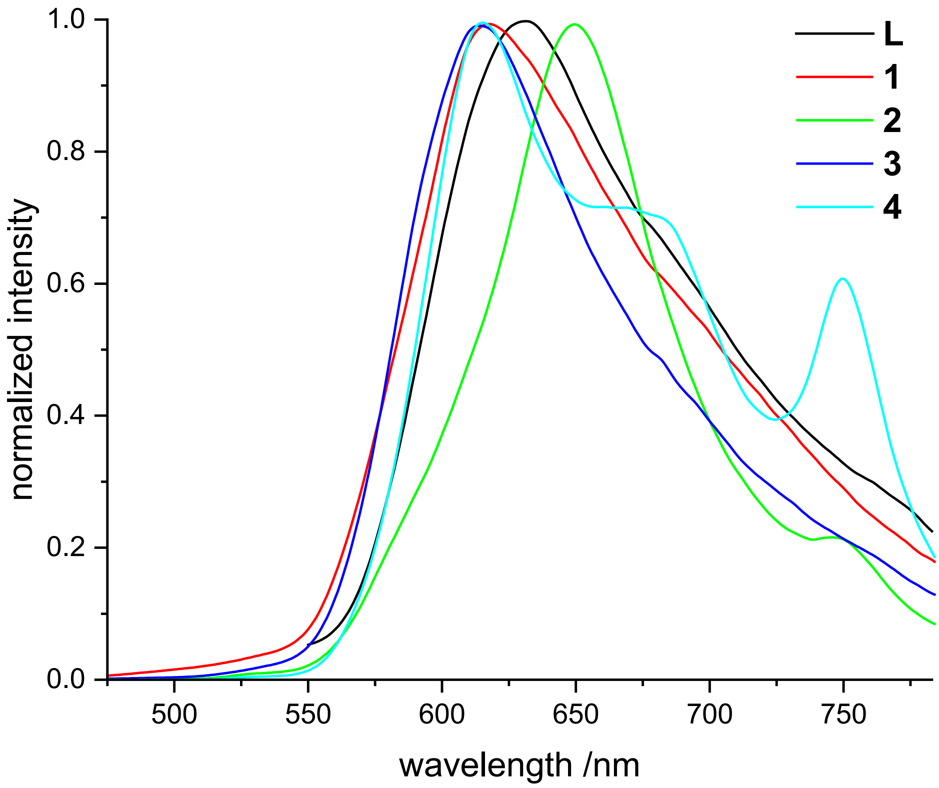

Figure S 5. PL spectra of **L** ($\lambda_{exc.}$ = 490 nm) and **1-4** ($\lambda_{exc.}$ = 405 nm).

Ligand L

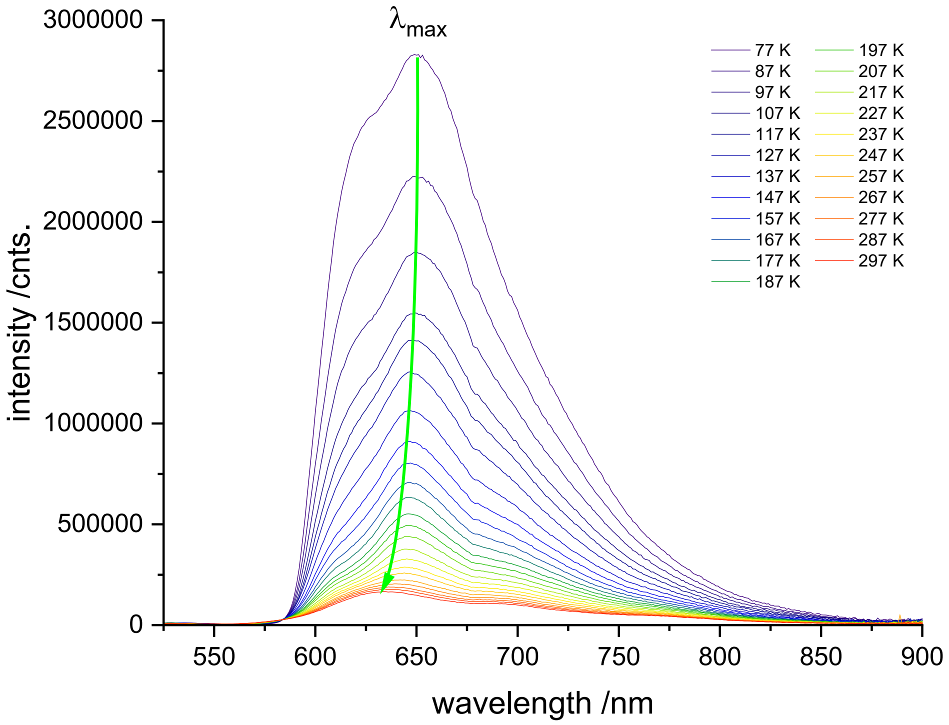

Figure S 6. Temperature-dependent PL spectra of **L** ($\lambda_{exc.}$ = 490 nm).

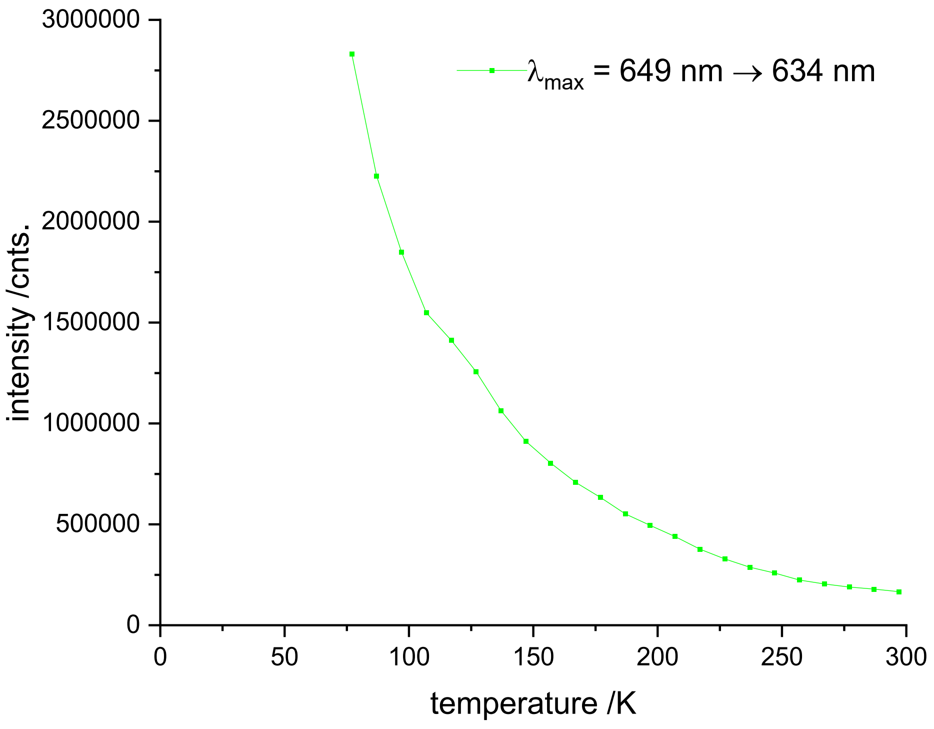

Figure S 7. Thermal evolution of the PL intensity of **L** at λ_{max}.

Coordination compound 1

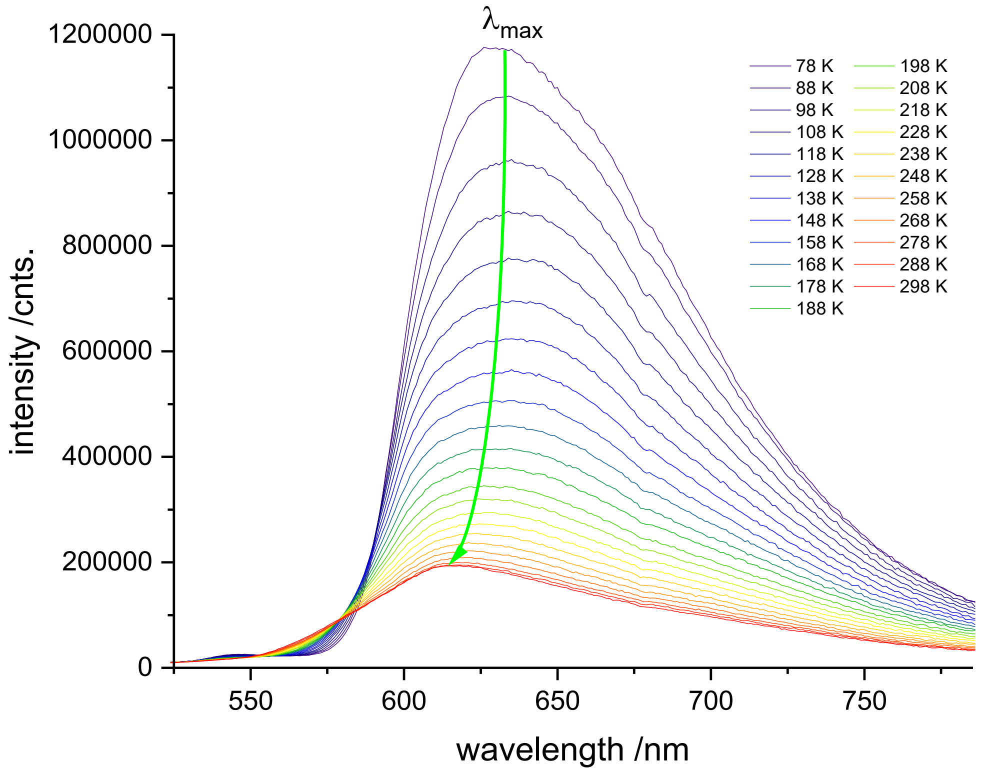

Figure S 8. Temperature-dependent PL spectra of **1** ($\lambda_{exc.}$ = 405 nm).

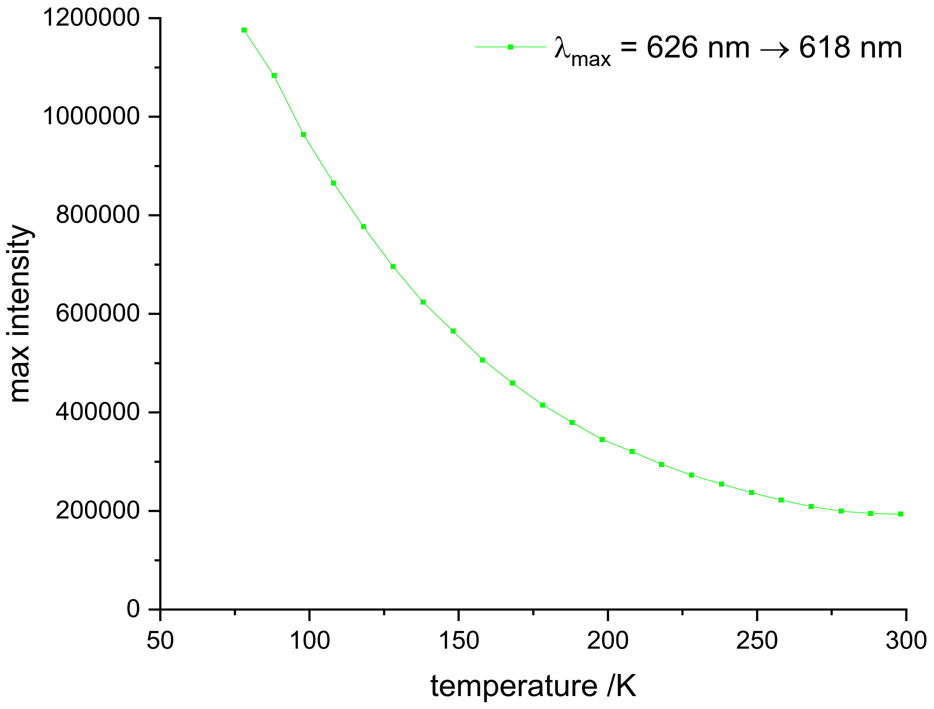

Figure S 9. Thermal evolution of the PL intensity of **1** at λ_{max}.

Coordination compound 2

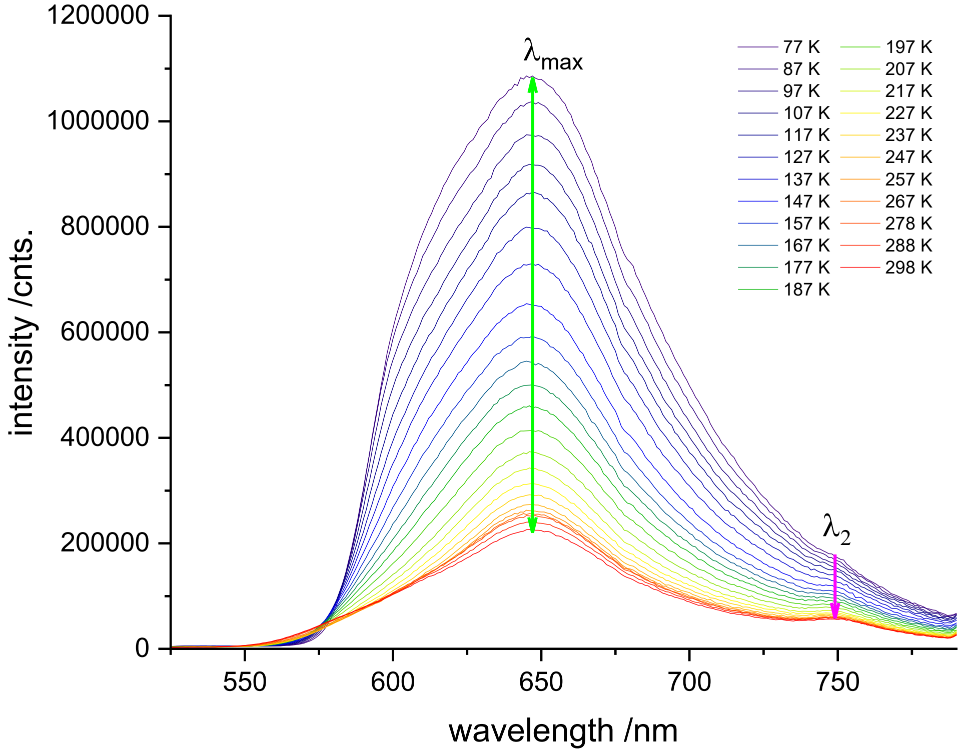

Figure S 10. Temperature-dependent PL spectra of **2** ($\lambda_{exc.}$ = 405 nm).

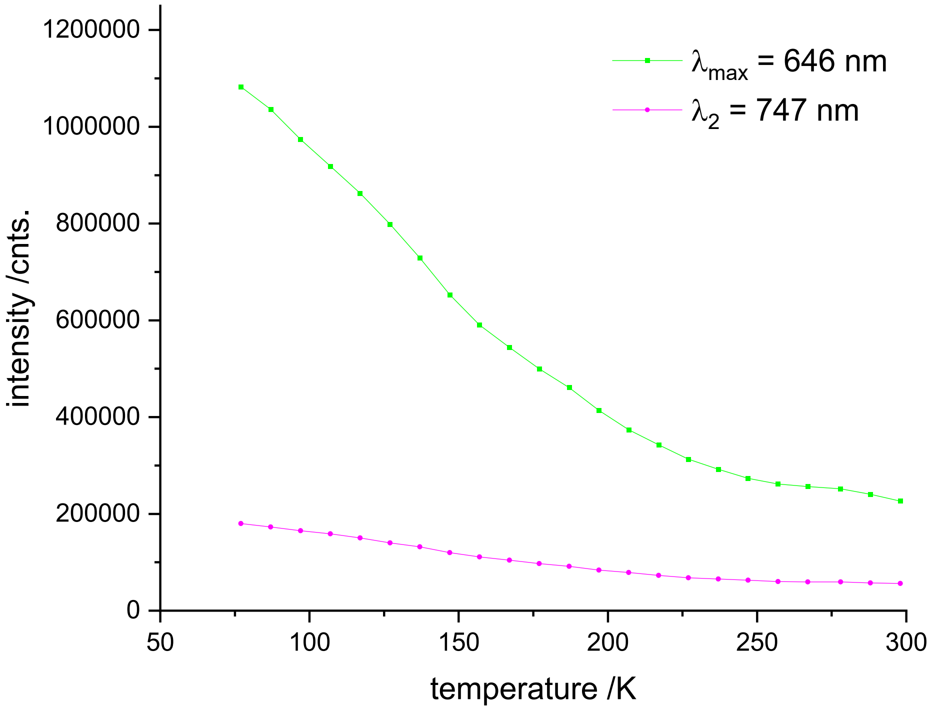

Figure S 11 Thermal evolution of the PL intensity of **2** at λ_{max} and λ_2.

Coordination compound 3

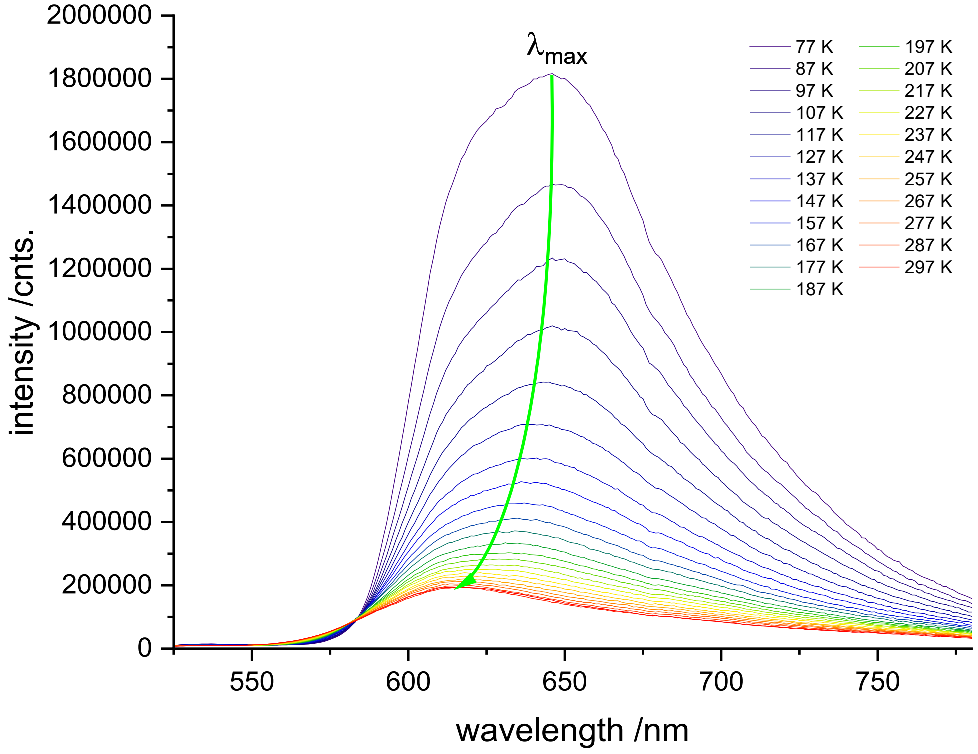

Figure S 12. Temperature-dependent PL spectra of **3** (**$\lambda_{exc.}$** = 405 nm).

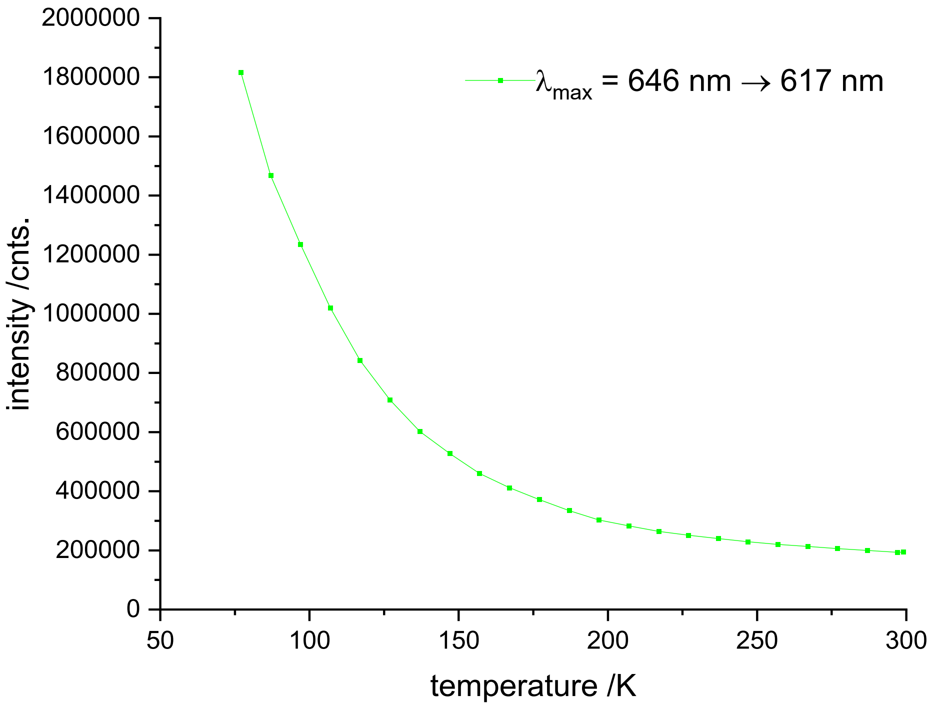

Figure S 13. Thermal evolution of the PL intensity of **3** at λ_{max}.

Coordination compound 4

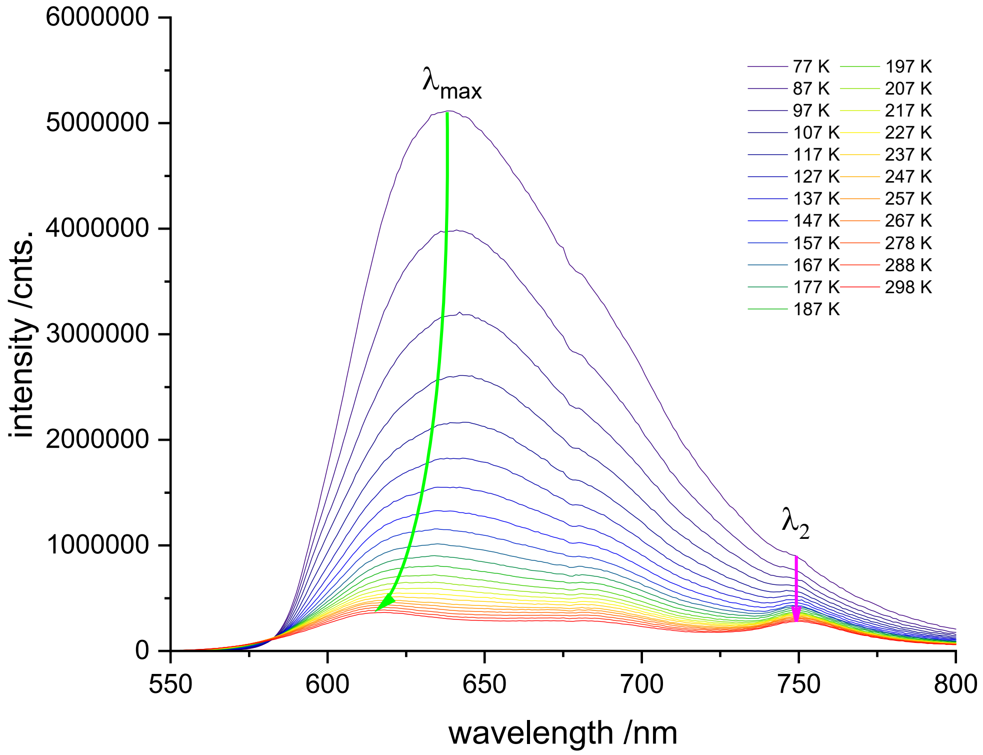

Figure S 14 Temperature-dependent PL spectra of **4** ($\lambda_{exc.}$ = 405 nm).

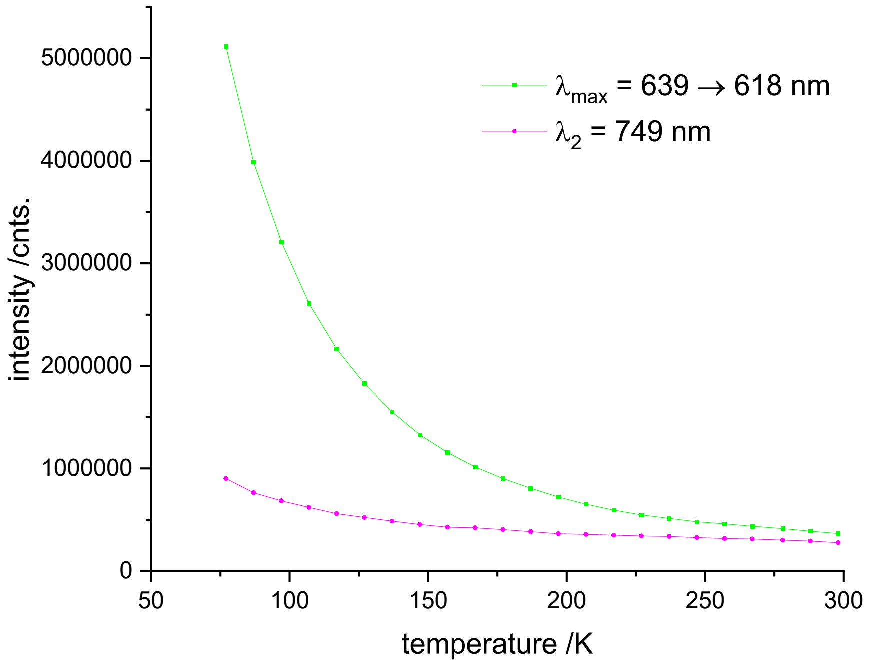

Figure S 15. Thermal evolution of the PL intensity of **4** at λ_{max} and λ_2.

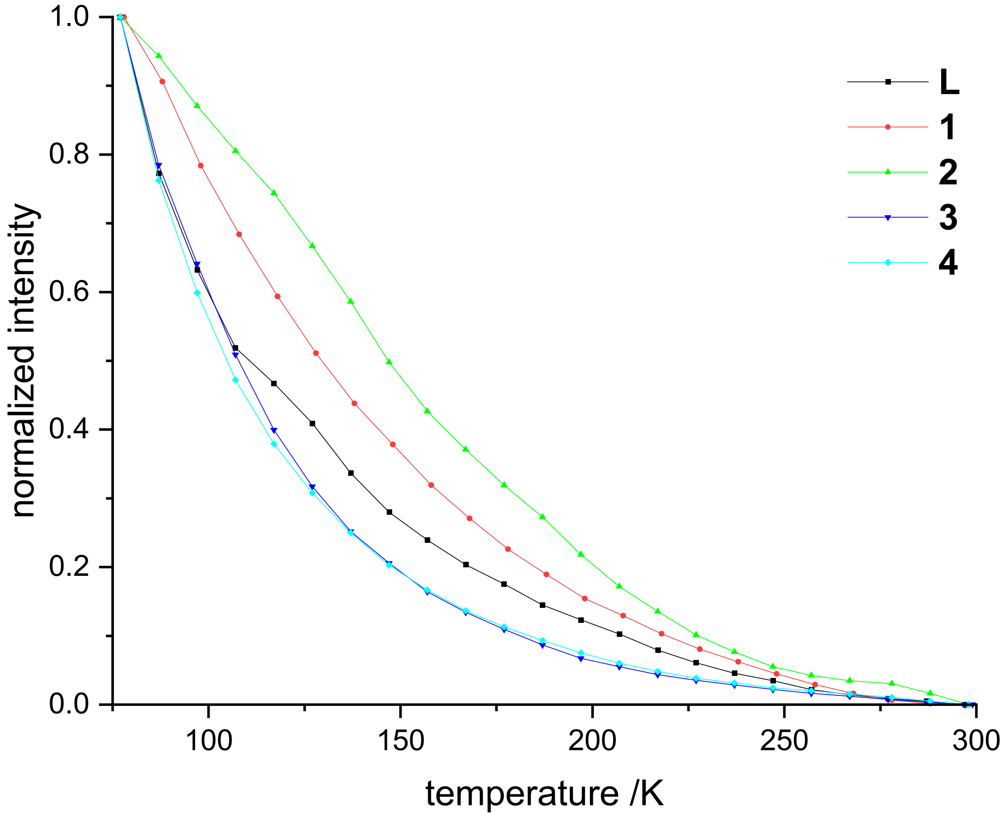

Figure S 16. Comparison of the thermal evolution of the PL intensity at λ_{max} for **L** and **1-4**.

XRPD measurements

Powder X-ray diffraction experiments were performed on an “Empyrean” (Panalytical) diffractometer using Cu K-α radiation, a primary beam filter (Bragg-Brentano HD), a fixed ½° divergence slit, a 0.04 rad soller slit and a GaliPIX3D detector. Finely powdered bulk samples were positioned on a silicon single crystal cut along the (711) plane. It was measured from 3° to 45° 2 θ with a step size of 0.014°.
Diffractograms were evaluated using the PANalytical program suite HighScorePlus [2]; background correction was applied, and a calculated diffraction pattern derived from single-crystal CIF data was subsequently refined via Rietveld analysis against the measured PXRD data to confirm phase purity and structural consistency.

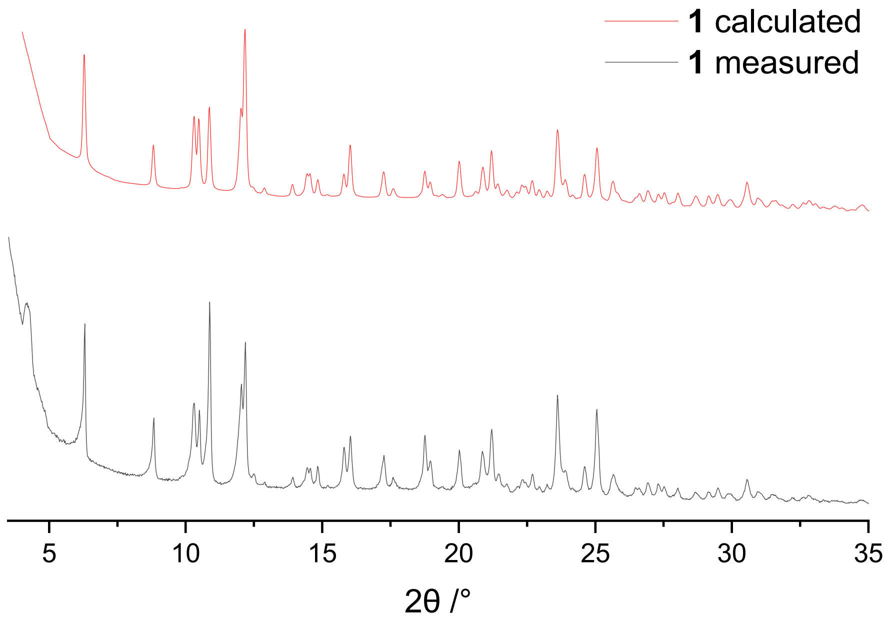

1 calculated
1 measured
5
10
15
20
25
30
35
2θ /°

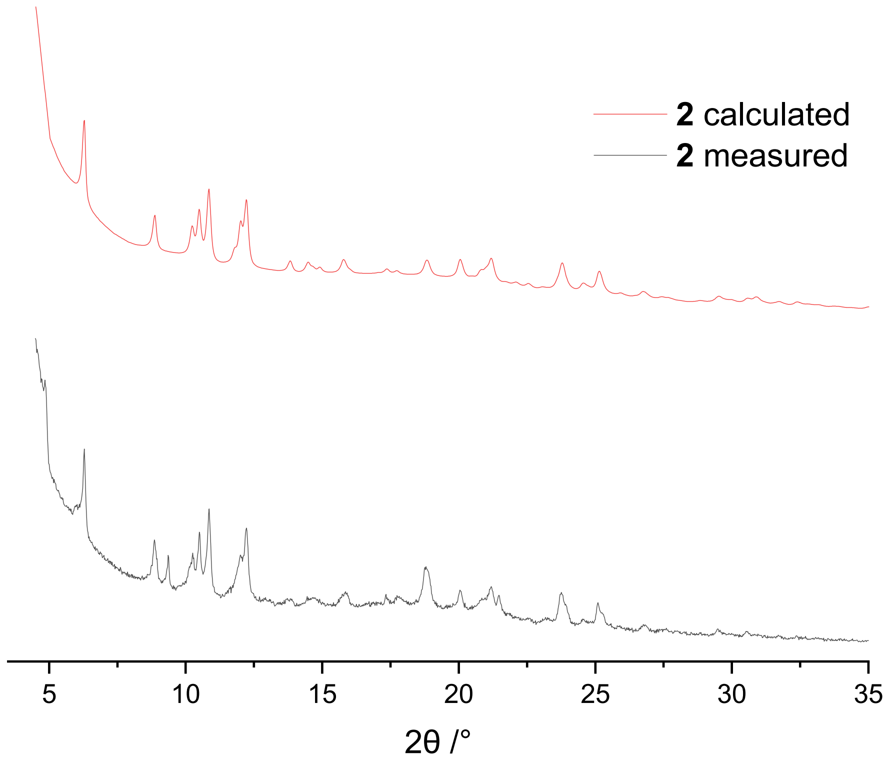

2 calculated
2 measured
5
10
15
20
25
30
35
2θ /°

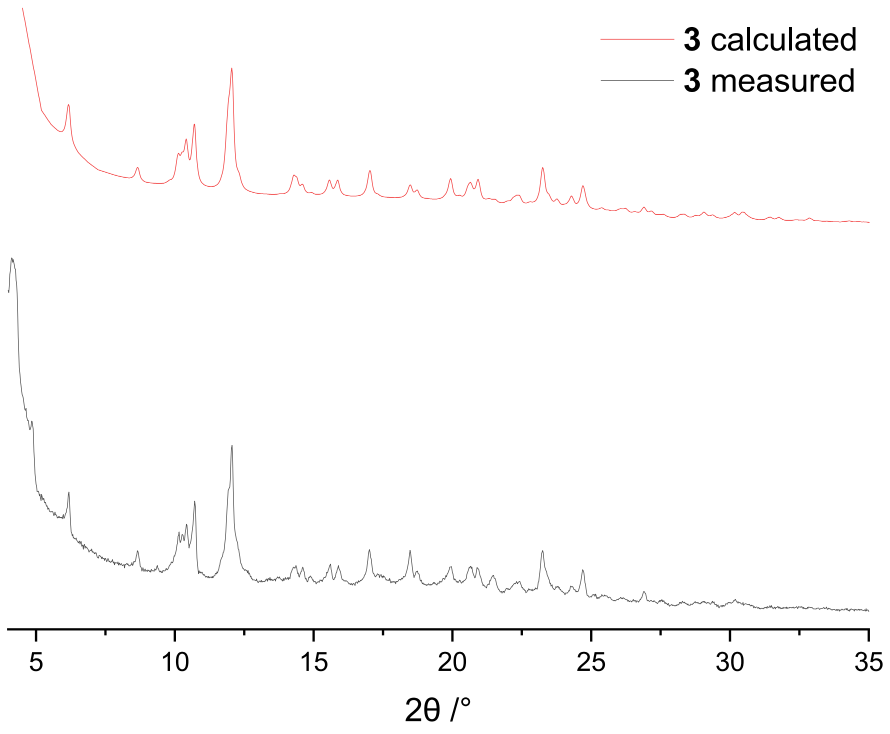
3 calculated
3 measured
5
10
15
20
25
30
35
2θ /°

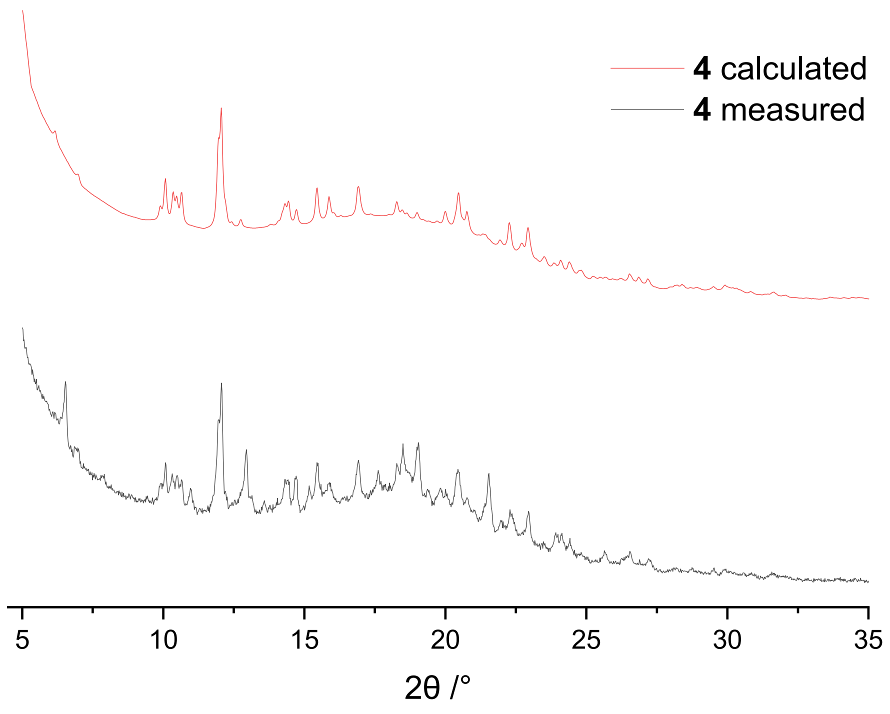
4 calculated
4 measured
5
10
15
20
25
30
35
2θ /°

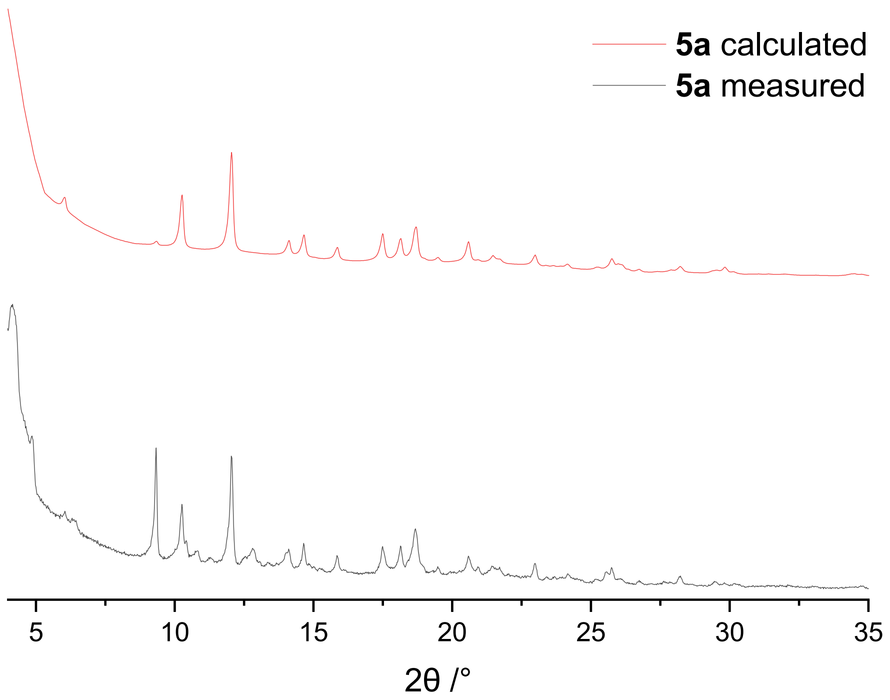
5a calculated
5a measured
5
10
15
20
25
30
35
2θ /°

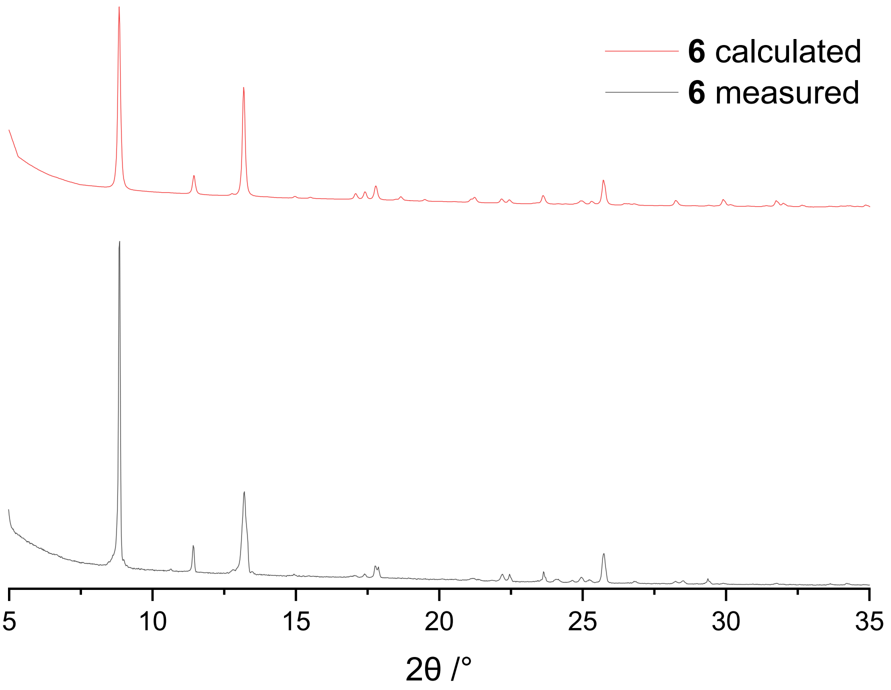
6 calculated
6 measured
5
10
15
20
25
30
35
2θ /°

X-Ray Structure Determination

X-ray diffraction data of **1** (CSD 2440404, 2440405) were collected at in a dry stream of nitrogen on a Bruker Kappa APEX II diffractometer system using Mo-*K*α radiation (λ = 0.71073 Å). Data were reduced to intensity values with SAINT and an absorption correction was applied with the multi-scan approach implemented in SADABS (Bruker computer programs: APEX3, SAINT and SADABS; Bruker AXS Inc., Madison, WI, 2020). Data of **2** (CSD 2440406, 2440407), **3** (CSD 2440408, 2440409), **4** (CSD 2440425–2440427), **5a** (CSD 2440428, 2440429), **5b** (CSD 2440430) and **6** (CSD 2440431) were collected in a dry stream of nitrogen on a STOE STADIVARI diffractometer system equipped with a Dectris Eiger CdTe hybrid photon counting detector, generally using Cu-*K*α radiation (λ = 1.54186 Å). To better resolve disorder, data of **5a** was collected using Mo-*K*α radiation (λ = 0.71073 Å). Data were reduced with X-Area or Integrate3d and an absorption correction was applied with the multi-scan approach implemented in LANA (X-Area 1.31.175.0, LANA 2.6.2.0; STOE & Cie GmbH, Darmstadt, Germany, 2021). The structures were solved by the dual-space approach implemented in SHELXT[3] and refined against F^2 with SHELXL[4]. For the refinement of the 180 K structure of **5a**, the $P2_1/c$ setting was chosen over the $P2_1/n$ setting with a β angle closer to 90° to simplify comparison with the remaining structures. The 100 K structure of **5a** was refined against two-domain intensity data with overlap information (HKLF5 format). Here likewise a non-reduced setting was used to simplify visual comparison with the 180 K phase. Molecular graphics were generated with the program MERCURY.[5]

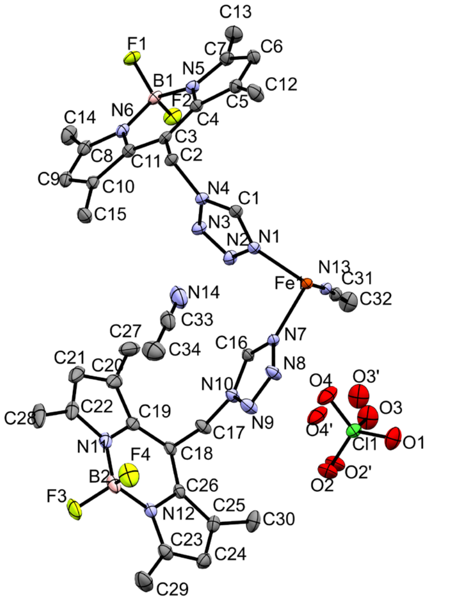

Figure S 17. Asymmetric unit of coordination compound **1** with atomic number labeling, showing the two different conformers of **L** (ellipsoids: 50 % probability level; atom color code: pink…B, grey…C, blue…N, red…O, light green…F green…Cl, orange…Fe; H-atoms are omitted for clarity).

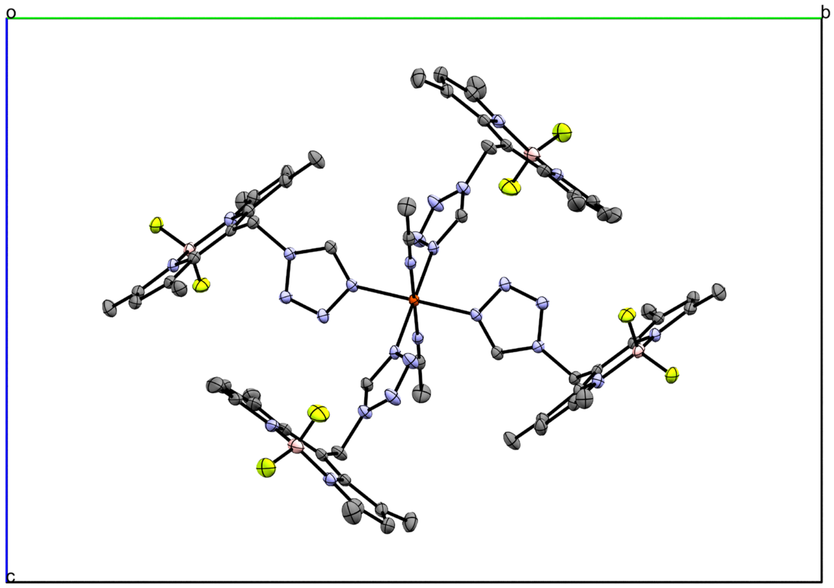

Figure S 18. Molecular structure of the mononuclear complex of coordination compound **1** at 100 K, viewed along the crystallographic *a*-axis (ellipsoids: 50 % probability level; atom color code: pink…B, grey…C, blue…N, light green…F, orange…Fe; H-atoms are omitted for clarity).

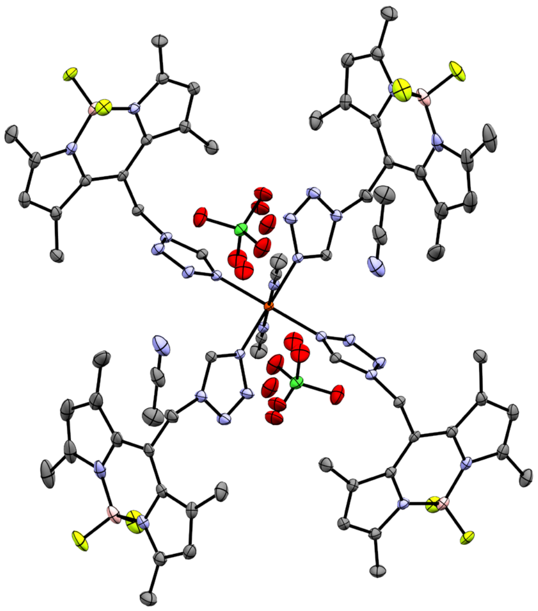

Figure S 19. Structure of coordination compound **1** at 100 K, showing the relative position of the solvate molecules (CH_3CN) and the anion molecules (ClO_4^-) (ellipsoids: 50 % probability level; atom color code: pink…B, grey…C, blue…N, red…O, light green…F, green…Cl, orange…Fe; H-atoms omitted for clarity).

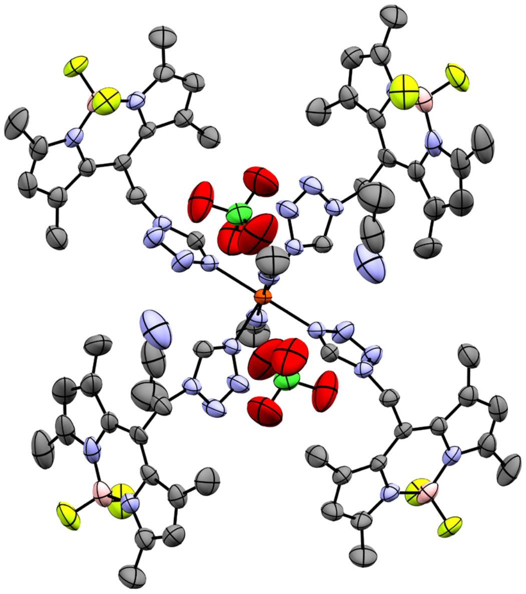

Figure S 20. Structure of coordination compound **1** at 300 K, showing the relative position of the solvate molecules (CH_3CN) and the anion molecules (ClO_4^-) (ellipsoids: 50 % probability level; atom color code: pink…B, grey…C, blue…N, red…O, light green…F, green…Cl, orange…Fe; H-atoms omitted for clarity).

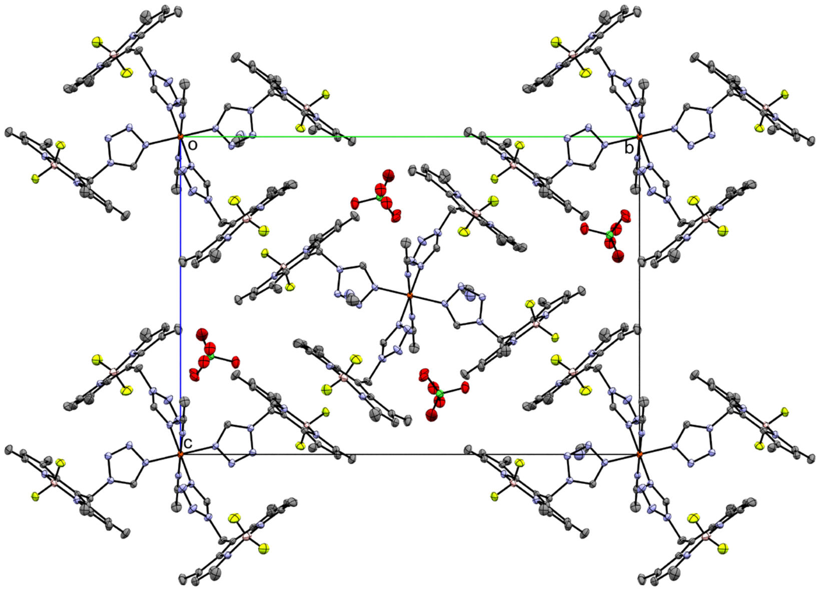

Figure S 21. Crystal packing of coordination compound **1** at 100 K, viewed along the crystallographic *a*-axis (ellipsoids: 50 % probability level; atom color code: pink…B, grey…C, blue…N, red…O, light green…F, green…Cl, orange…Fe; H-atoms omitted for clarity).

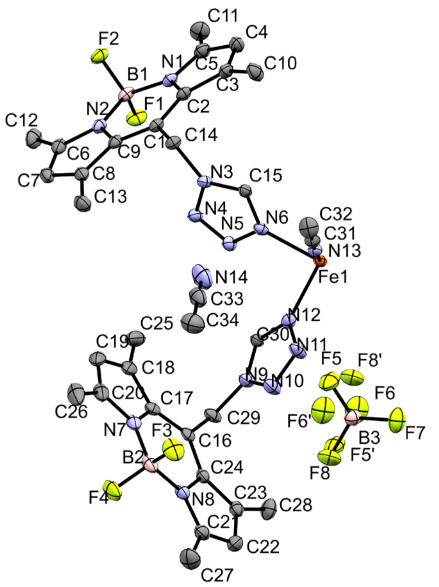

Figure S 22. Asymmetric unit of coordination compound **2** with atomic number labeling, showing the two different conformers of **L** (ellipsoids: 50 % probability level; atom color code: pink…B, grey…C, blue…N, light green…F, orange…Fe; H-atoms are omitted for clarity).

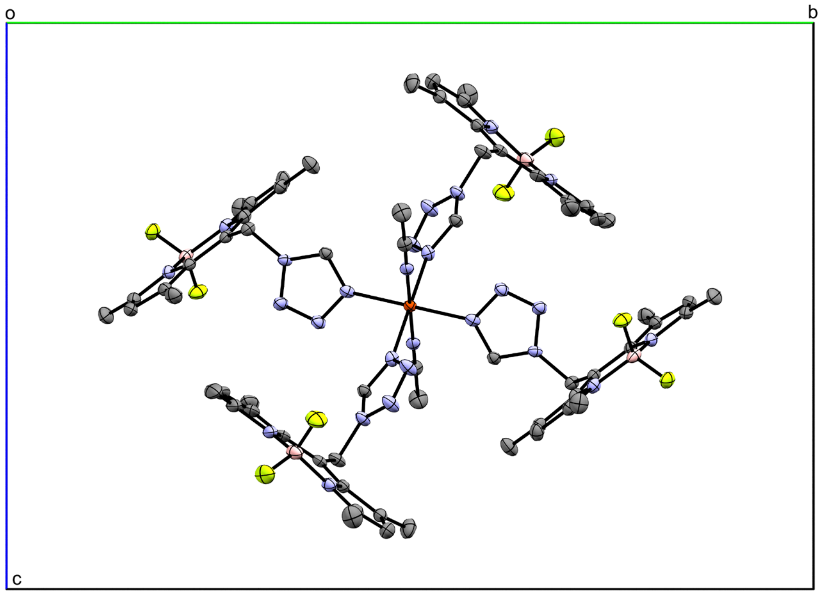

Figure S 23. Molecular structure of the mononuclear complex of coordination compound **2** at 100 K, viewed along the crystallographic *a*-axis (ellipsoids: 50 % probability level; atom color code: pink…B, grey…C, blue…N, light green…F, orange…Fe; H-atoms are omitted for clarity).

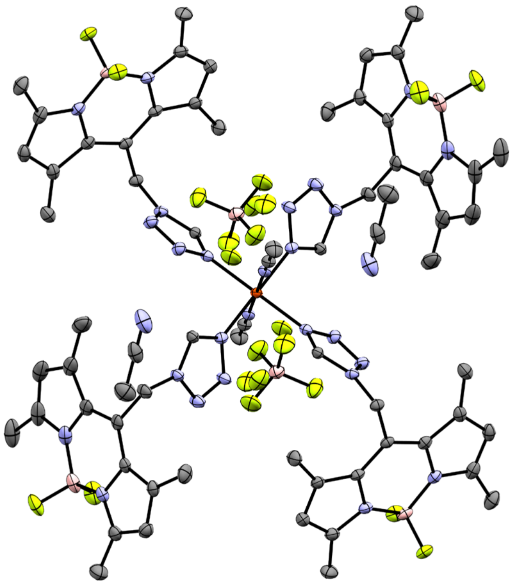

Figure S 24. Structure of coordination compound **2** at 100 K, showing the relative position of the solvate molecules (CH_3CN) and the anion molecules (BF_4^-) (ellipsoids: 50 % probability level; atom color code: pink…B, grey…C, blue…N, light green…F, orange…Fe; H-atoms omitted for clarity).

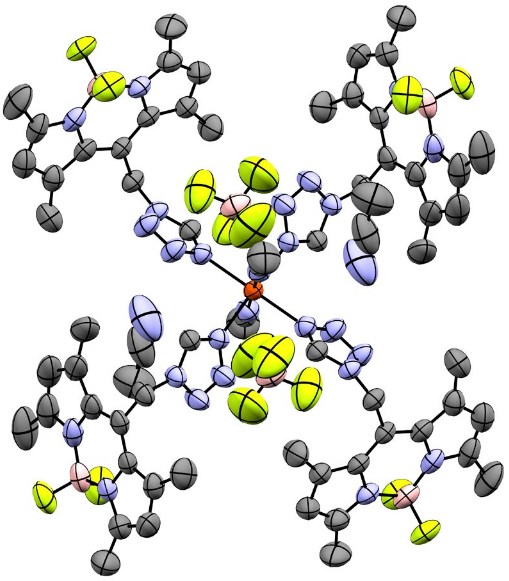

Figure S 25. Structure of coordination compound **2** at 300 K, showing the relative position of the solvate molecules (CH_3CN) and the anion molecules (BF_4^-) (ellipsoids: 50 % probability level; atom color code: pink…B, grey…C, blue…N, light green…F, orange…Fe; H-atoms omitted for clarity).

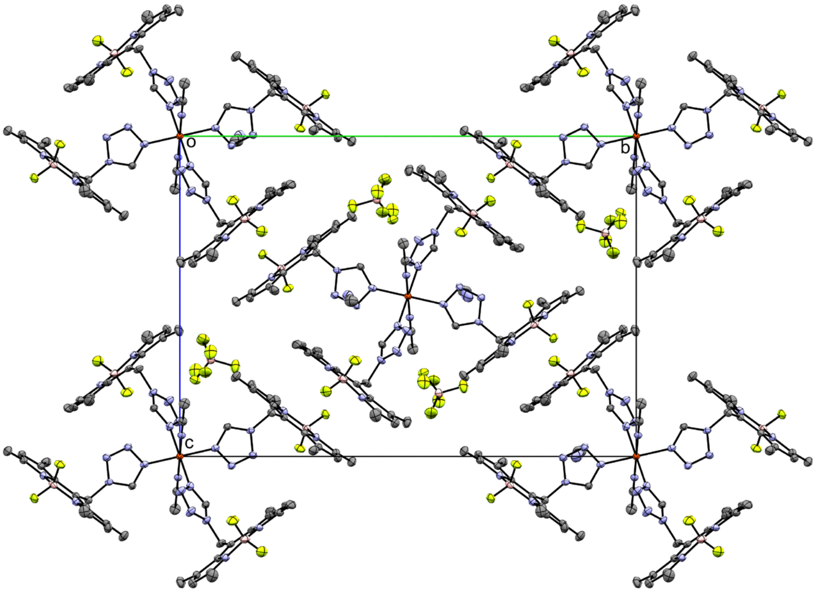

Figure S 26. Crystal packing of coordination compound **2** at 100 K, viewed along the crystallographic *a*-axis (ellipsoids: 50 % probability level; atom color code: pink…B, grey…C, blue…N, light green…F, orange…Fe; H-atoms omitted for clarity).

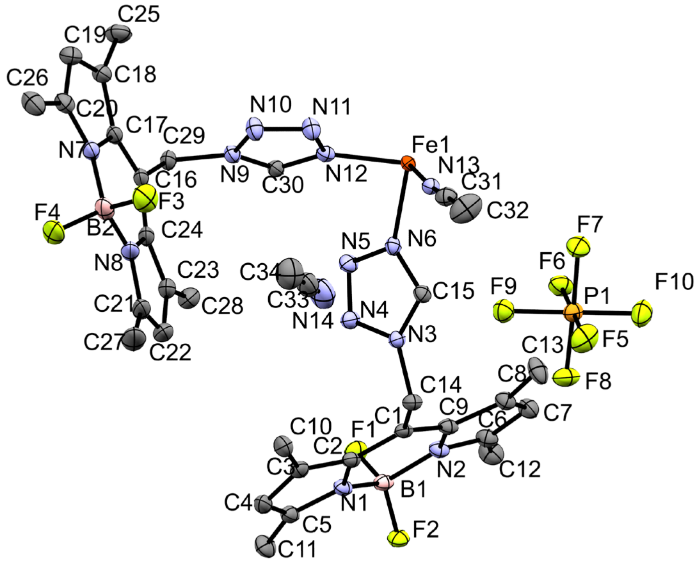

Figure S 27. Asymmetric unit of coordination compound **3** with atomic number labeling, showing the two different conformers of **L** (ellipsoids: 50 % probability level; atom color code: pink…B, grey…C, blue…N, light green…F, orange…Fe, tangerine…P; H-atoms are omitted for clarity).

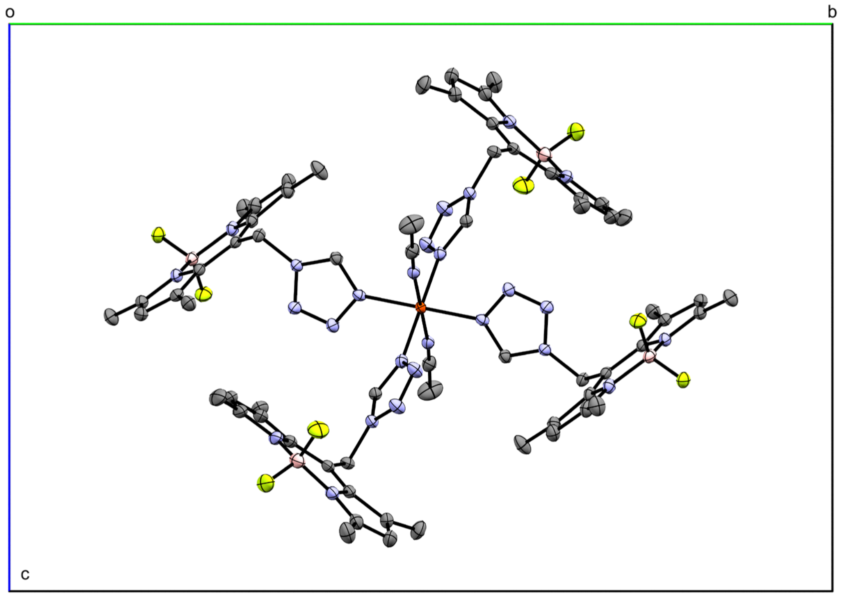

Figure S 28. Molecular structure of the mononuclear complex of coordination compound **3** at 100 K, viewed along the crystallographic *a*-axis (ellipsoids: 50 % probability level; atom color code: pink…B, grey…C, blue…N, light green…F, orange…Fe; H-atoms are omitted for clarity).

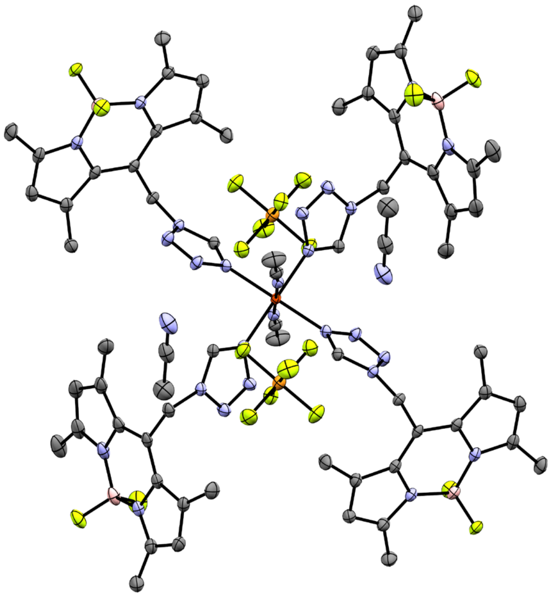

Figure S 29. Structure of coordination compound **3** at 100 K, showing the relative position of the solvate molecules (CH_3CN) and the anion molecules (PF_6^-) (ellipsoids: 50 % probability level; atom color code: pink…B, grey…C, blue…N, light green…F, orange…Fe, tangerine…P; H-atoms omitted for clarity).

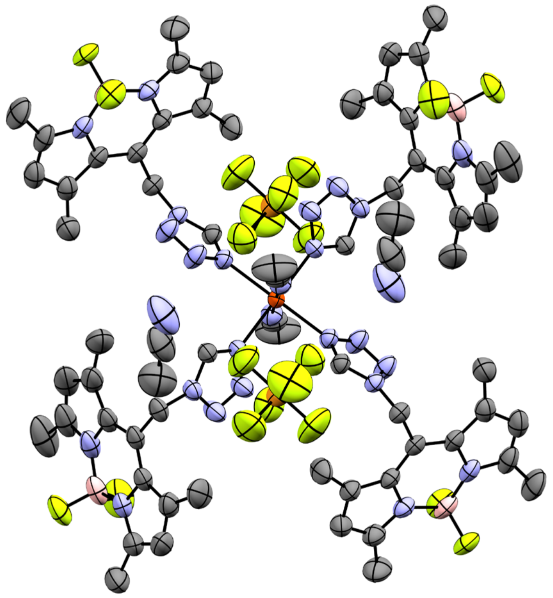

Figure S 30. Structure of coordination compound **3** at 300 K, showing the relative position of the solvate molecules (CH_3CN) and the anion molecules (PF_6^-) (ellipsoids: 50 % probability level; atom color code: pink…B, grey…C, blue…N, light green…F, orange…Fe, tangerine…P; H-atoms omitted for clarity).

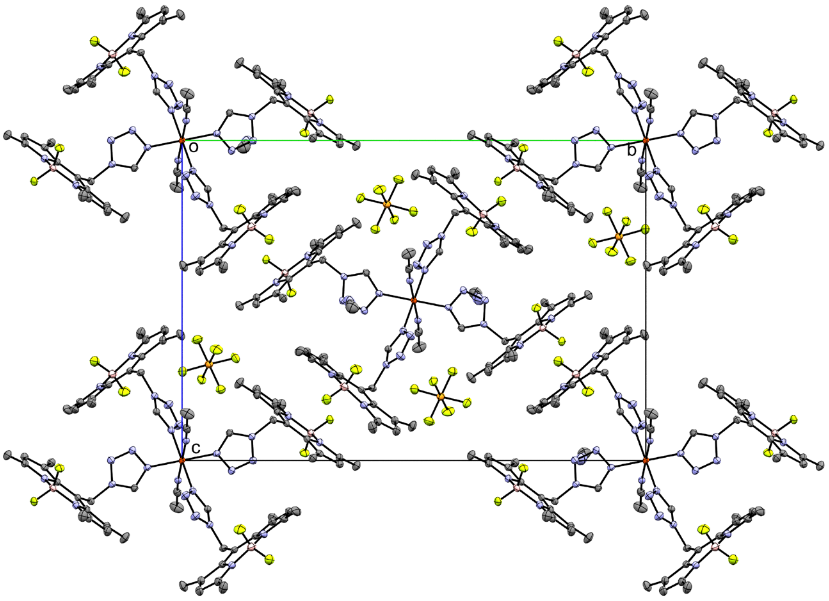

Figure S 31. Crystal packing of coordination compound **3** at 100 K, viewed along the crystallographic *a*-axis (ellipsoids: 50 % probability level; atom color code: pink…B, grey…C, blue…N, light green…F, orange…Fe, tangerine…P; H-atoms omitted for clarity).

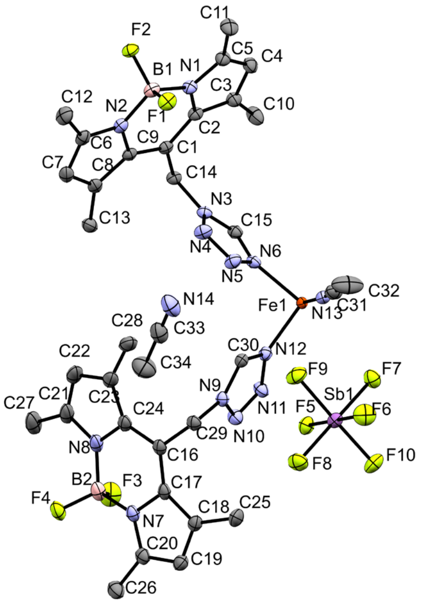

Figure S 32. Asymmetric unit of coordination compound **4** with atomic number labeling, showing the two different conformers of **L** (ellipsoids: 50 % probability level; atom color code: pink…B, grey…C, blue…N, light green…F, orange…Fe, amethyst…Sb; H-atoms are omitted for clarity).

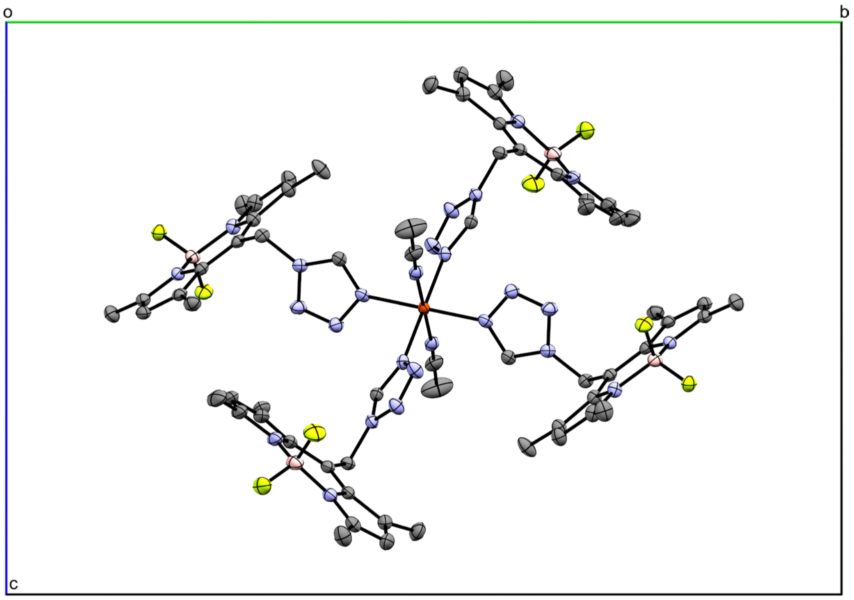

Figure S 33. Molecular structure of the mononuclear complex of coordination compound **4** at 90 K, viewed along the crystallographic *a*-axis (ellipsoids: 50 % probability level; atom color code: pink…B, grey…C, blue…N, light green…F, orange…Fe; H-atoms are omitted for clarity).

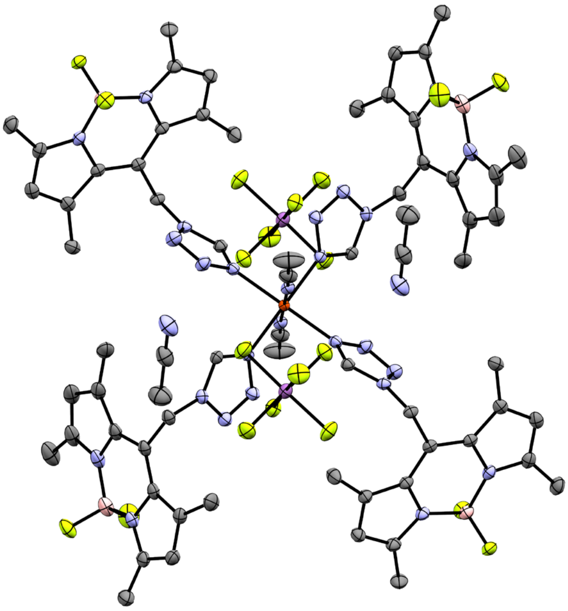

Figure S 34. Structure of coordination compound **4** at 90 K, showing the relative position of the solvate molecules (CH_3CN) and the anion molecules (SbF_6^-) (ellipsoids: 50 % probability level; atom color code: pink…B, grey…C, blue…N, light green…F, orange…Fe, amethyst…Sb; H-atoms omitted for clarity).

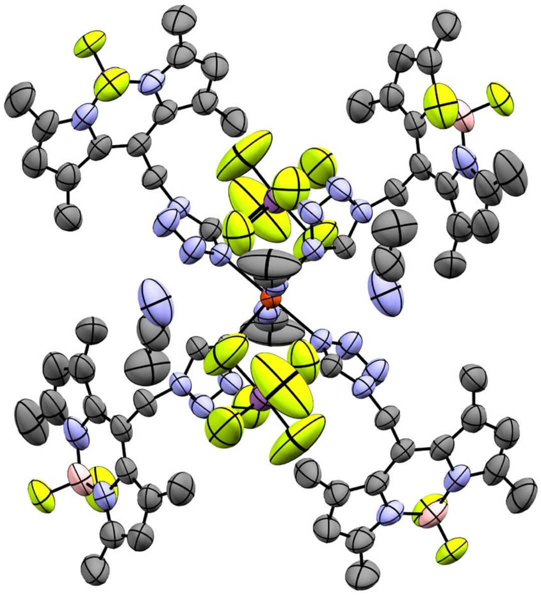

Figure S 35. Structure of coordination compound **4** at 300 K, showing the relative position of the solvate molecules (CH_3CN) and the anion molecules (SbF_6^-) (ellipsoids: 50 % probability level; atom color code: pink…B, grey…C, blue…N, light green…F, orange…Fe, amethyst…Sb; H-atoms omitted for clarity).

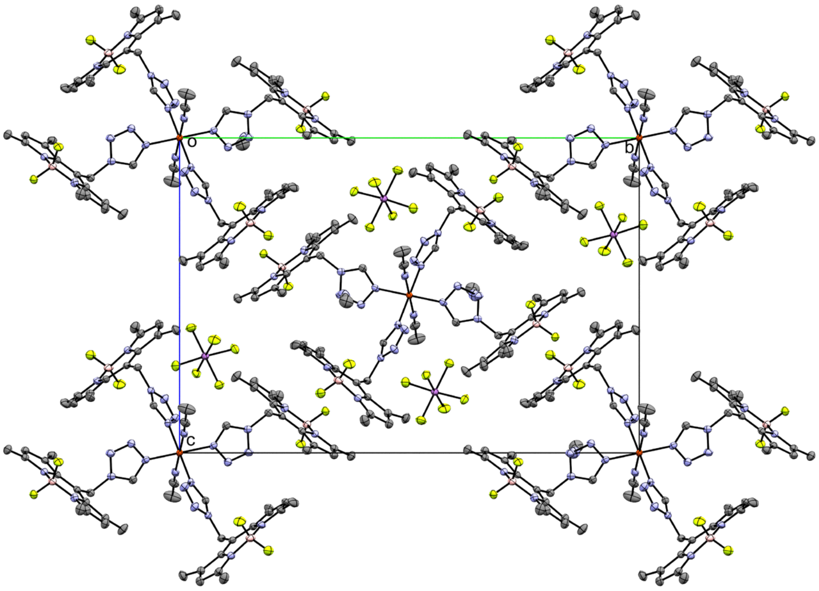

Figure S 36. Crystal packing of coordination compound **4** at 90 K, viewed along the crystallographic *a*-axis (ellipsoids: 50 % probability level; atom color code: pink…B, grey…C, blue…N, light green…F, orange…Fe, amethyst…Sb; H-atoms omitted for clarity).

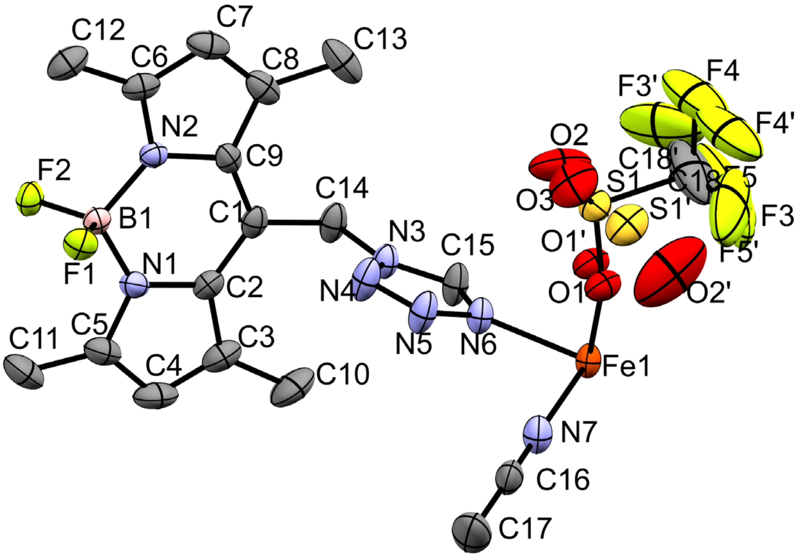

Figure S 37. Asymmetric unit of coordination compound **5a** with atomic number labeling, showing the coordinating anion ($CF_3SO_3^-$) (ellipsoids: 50 % probability level; atom color code: pink…B, grey…C, blue…N, red…O, yellow…S, light green…F, orange…Fe; H-atoms are omitted for clarity).

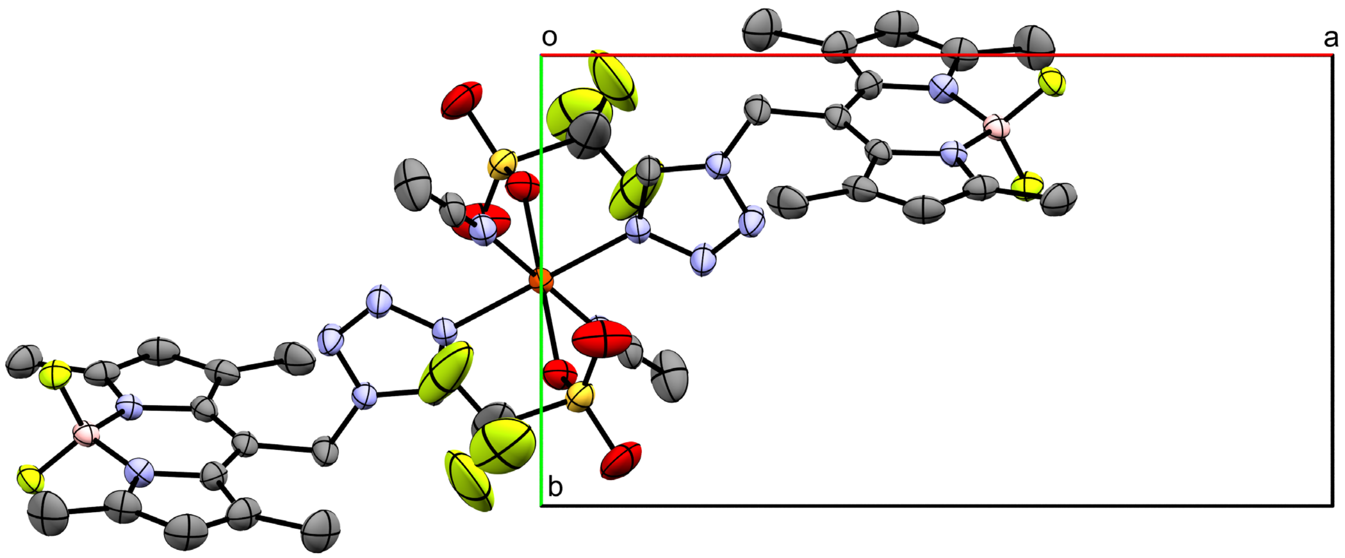

Figure S 38. Molecular structure of the mononuclear complex of coordination compound **5a** at 180 K, viewed along the crystallographic *c*-axis (ellipsoids: 50 % probability level; atom color code: pink…B, grey…C, blue…N, red…O, yellow…S, light green…F, orange…Fe; H-atoms and minor positions of the disordered anion are omitted for clarity).

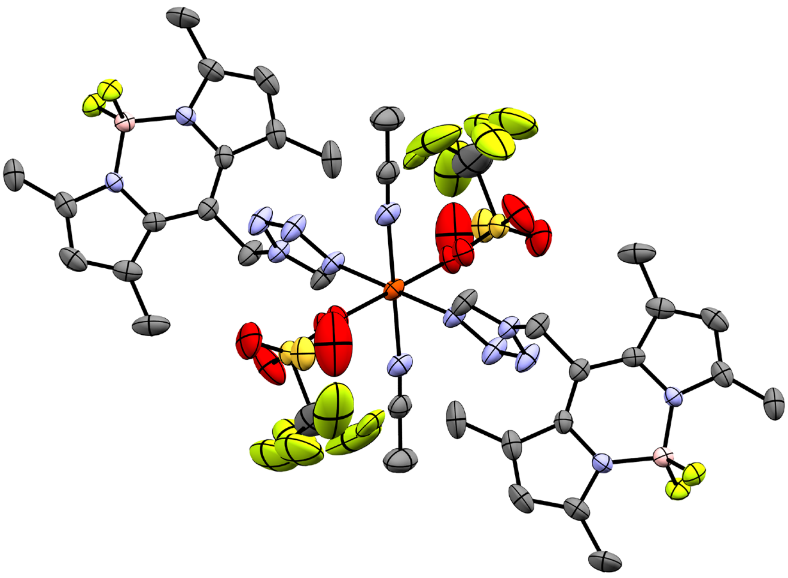

Figure S 39. Molecular structure of coordination compound **5a** at 180 K, featuring CH_3CN as the co-ligand and $CF_3SO_3^-$ as the coordinating anion, which is disordered (ellipsoids: 50 % probability level; atom color code: pink…B, grey…C, blue…N, light green…F, red…O, yellow…S, orange…Fe; H-atoms are omitted for clarity).

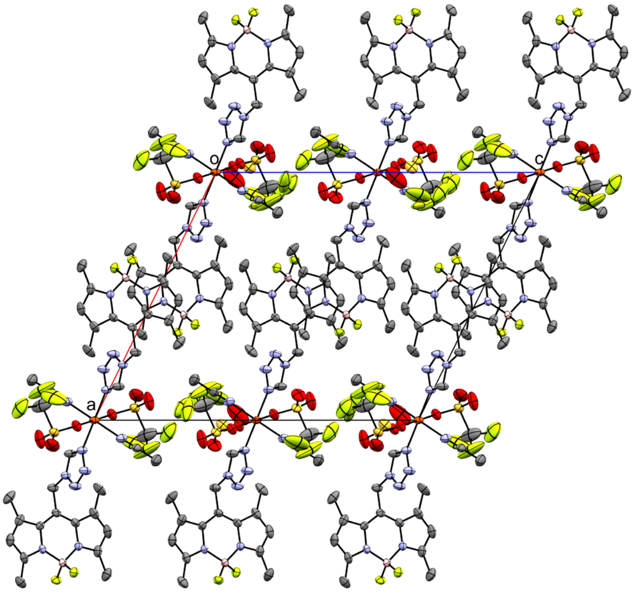

Figure S 40. Crystal packing of coordination compound **5a** at 180 K, viewed along the crystallographic *b*-axis (ellipsoids: 50 % probability level; atom color code: pink…B, grey…C, blue…N, light green…F, red…O, yellow…S, orange…Fe; H-atoms are omitted for clarity).

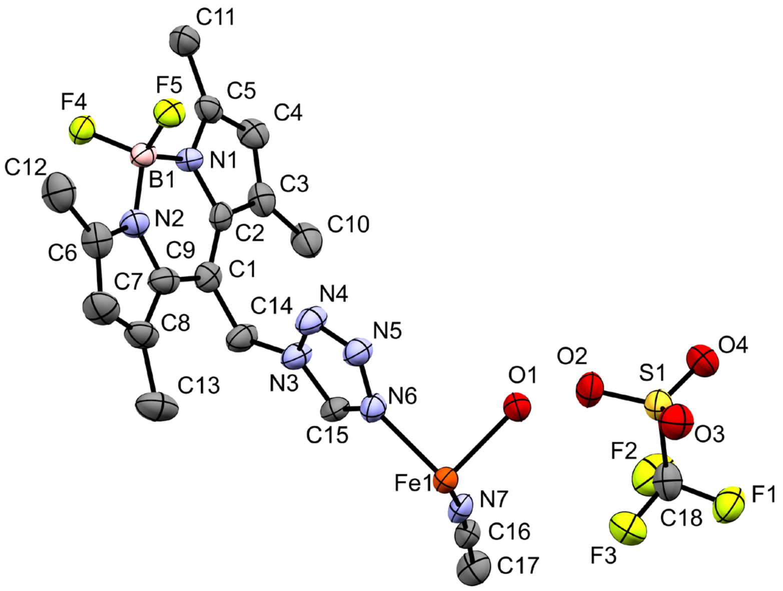

Figure S 41. Asymmetric unit of coordination compound **5b** with atomic number labeling, showing the non-coordinating anion ($CF_3SO_3^-$) and water as well as CH_3CN as co-ligand (ellipsoids: 50 % probability level; atom color code: pink…B, grey…C, blue…N, red…O, yellow…S, light green…F, orange…Fe; H-atoms are omitted for clarity).

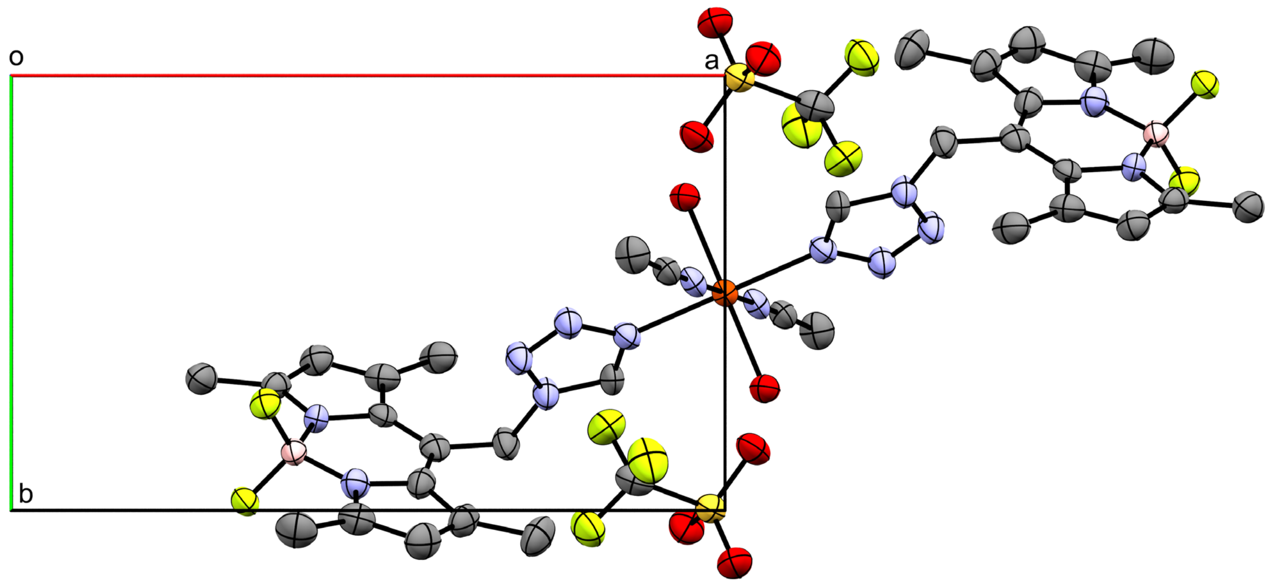

Figure S 42. Structure of the mononuclear complex of coordination compound **5b** at 100 K, viewed along the crystallographic *c*-axis (ellipsoids: 50 % probability level; atom color code: pink…B, grey…C, blue…N, red…O, yellow…S, light green…F, orange…Fe; H-atoms are omitted for clarity).

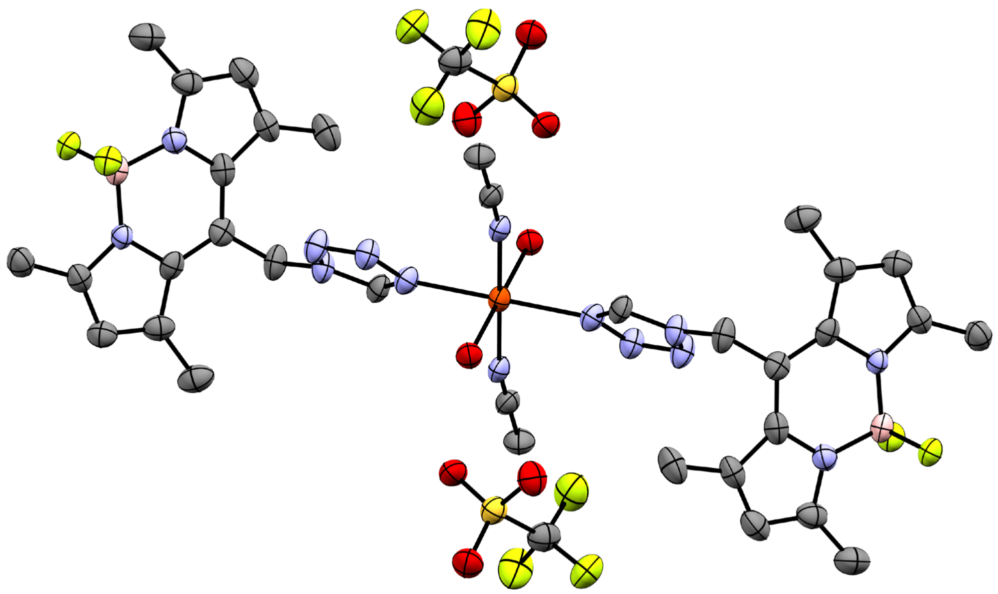

Figure S 43. Structure of coordination compound **5b** at 100 K, featuring CH_3CN and water as the co-ligand, and $CF_3SO_3^-$ as the non-coordinating anion (ellipsoids: 50 % probability level; atom color code: pink…B, grey…C, blue…N, light green…F, red…O, yellow…S, orange…Fe; H-atoms are omitted for clarity).

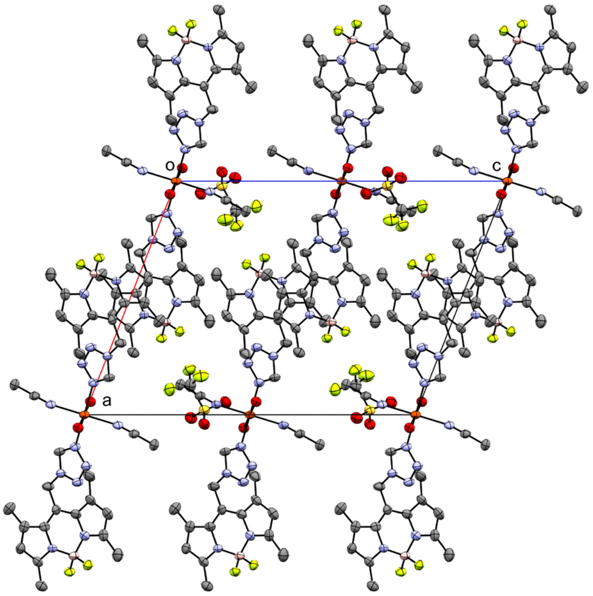

Figure S 44. Crystal packing of coordination compound **5b** at 100 K, viewed along the crystallographic *b*-axis (ellipsoids: 50 % probability level; atom color code: pink…B, grey…C, blue…N, light green…F, red…O, yellow…S, orange…Fe; H-atoms are omitted for clarity).

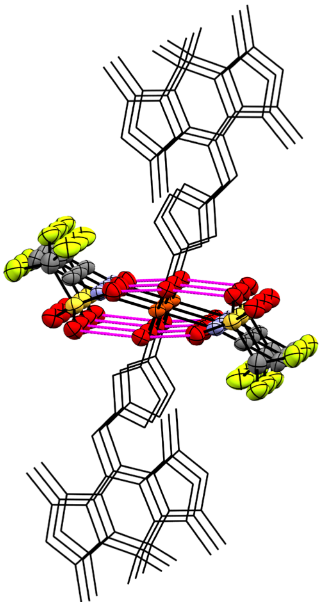

Figure S 45. H-bond network in **5b** at 100 K, viewed along the crystallographic *b*-axis, with H-bonds represented as magenta-dotted lines (ellipsoids: 50 % probability level atom color code: grey…C, blue…N, light green…F, red…O, yellow…S, orange…Fe; H-atoms are omitted for clarity and **L** molecules are depicted as wireframes for better visualization)

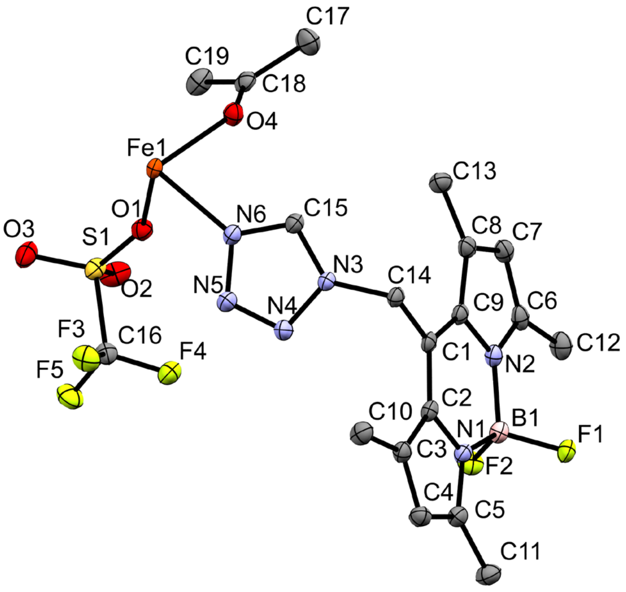

Figure S 46. Asymmetric unit of coordination compound **6** with atomic number labeling, showing the coordinating anion ($CF_3SO_3^-$) (ellipsoids: 50 % probability level; atom color code: pink…B, grey…C, blue…N, red…O, yellow…S, light green…F, orange…Fe; H-atoms are omitted for clarity).

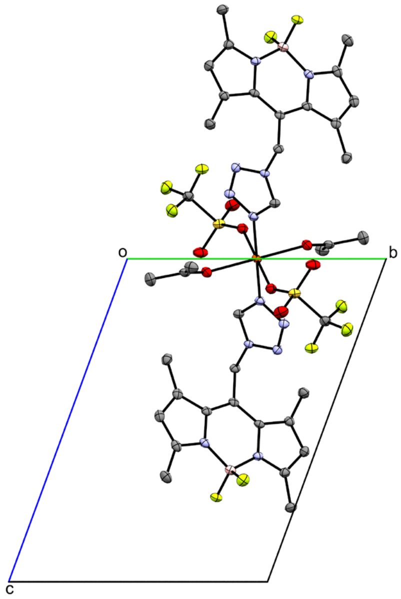

Figure S 47. Molecular structure of the mononuclear complex of coordination compound **6** at 100 K, viewed along the crystallographic *a*-axis (ellipsoids: 50 % probability level; atom color code: pink…B, grey…C, blue…N, red…O, yellow…S, light green…F, orange…Fe; H-atoms are omitted for clarity).

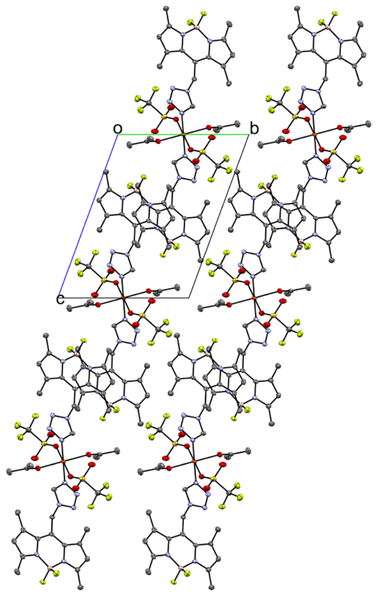

Figure S 48. Crystal packing of coordination compound **6** at 100 K, viewed along the crystallographic *a*-axis (ellipsoids: 50 % probability level; atom color code: pink…B, grey…C, blue…N, light green…F, red…O, yellow…S, orange…Fe; H-atoms are omitted for clarity).

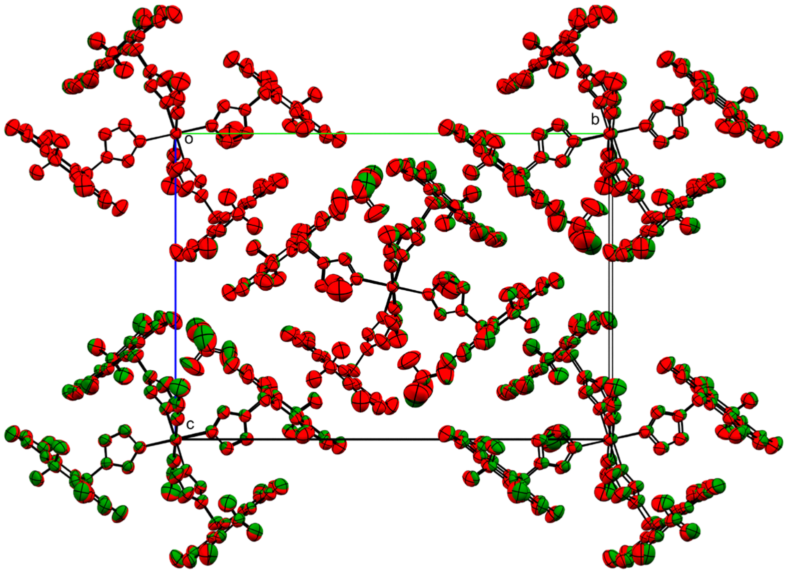

Figure S 49. Crystal packing comparison of coordination compound **1** (green) and **2** (red) at 300 K, showing hardly any differences (ellipsoids: 50 % probability level; H-atoms omitted for clarity).

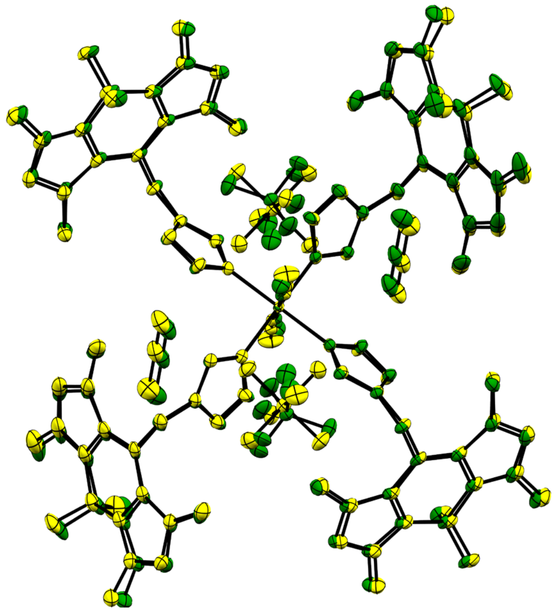

Figure S 50. Structure comparison of coordination compound **1** (green) and **3** (yellow) at 100 K, showing small differences in the spatial arrangement of the ligands, the anion molecules and the solvate molecules (ellipsoids: 50 % probability level; H-atoms omitted for clarity).

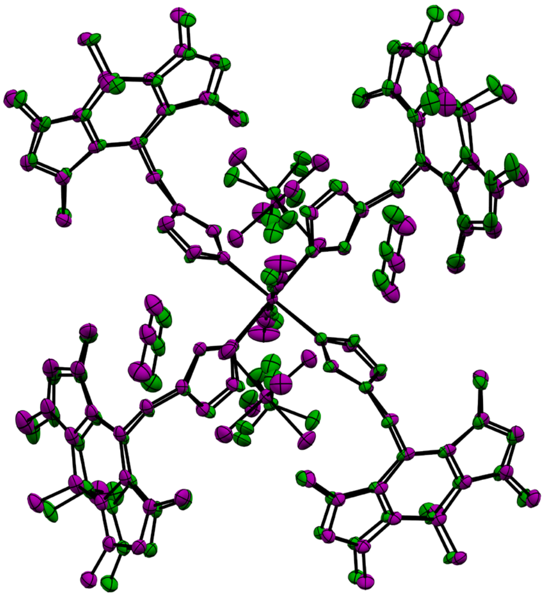

Figure S 51. Structure comparison of coordination compound **1** (green) and **4** (purple) at 100 K and 90 K, respectively, showing small differences in the spatial arrangement of the ligands, the anion molecules and the solvate molecules (ellipsoids: 50 % probability level; H-atoms omitted for clarity).

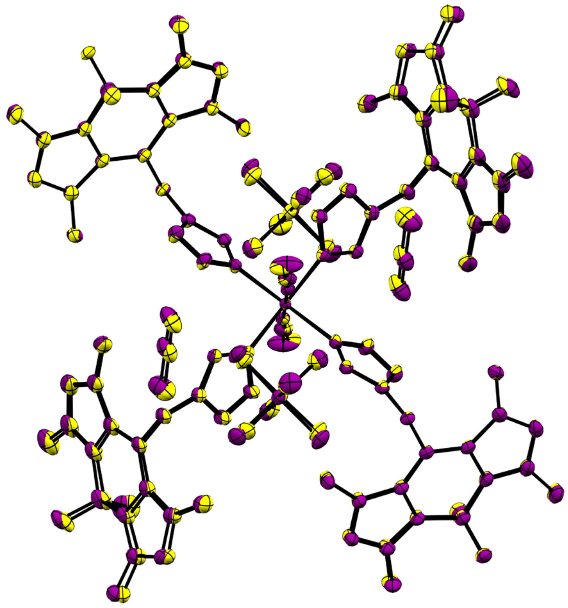

Figure S 52. Structure comparison of coordination compound **3** (yellow) and **4** (purple) at 100 K and 90 K, respectively, showing hardly any differences in the spatial arrangement of the ligands, the anion molecules and the solvate molecules (ellipsoids: 50 % probability level; H-atoms omitted for clarity).

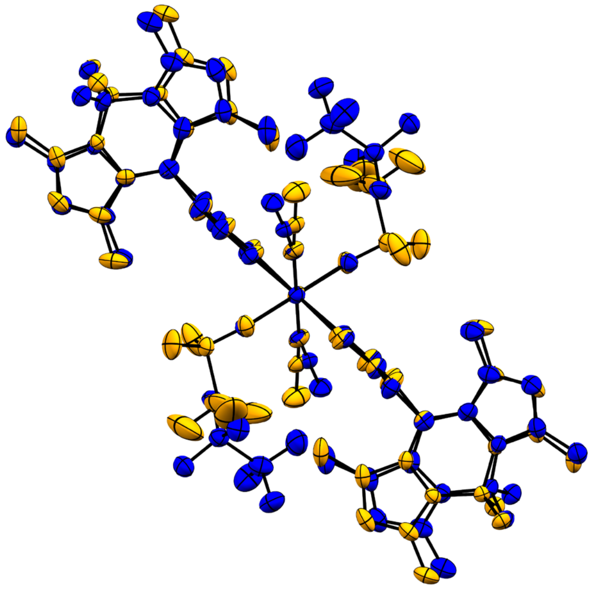

Figure S 53. Structure comparison of coordination compound **5a** (gold) and **5b** (blue) at 180 K and 100 K, respectively, showing small differences in the spatial arrangement of the ligands **L** and the co-ligand molecules (CH_3CN), as well as the anion ($CF_3SO_3^-$) in a coordinating mode in **5a** and a non-coordinating mode in **5b** (ellipsoids: 50 % probability level; H-atoms omitted for clarity).

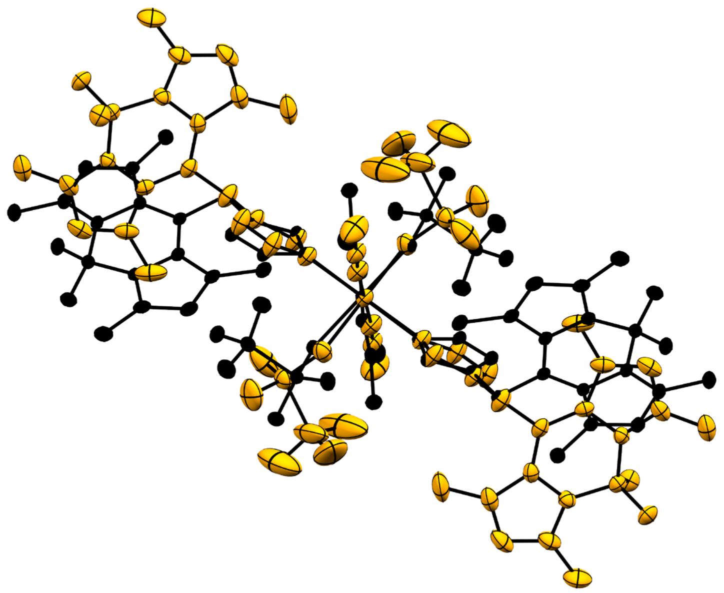

Figure S 54. Structure comparison of coordination compound **5a** (gold) and **6** (black) at 180 K and 100 K, respectively, showing differences in the spatial arrangement of the ligands **L**, the co-ligand molecules (CH_3CN and acetone), and the coordinating anion ($CF_3SO_3^-$) (ellipsoids: 50 % probability level; H-atoms omitted for clarity).

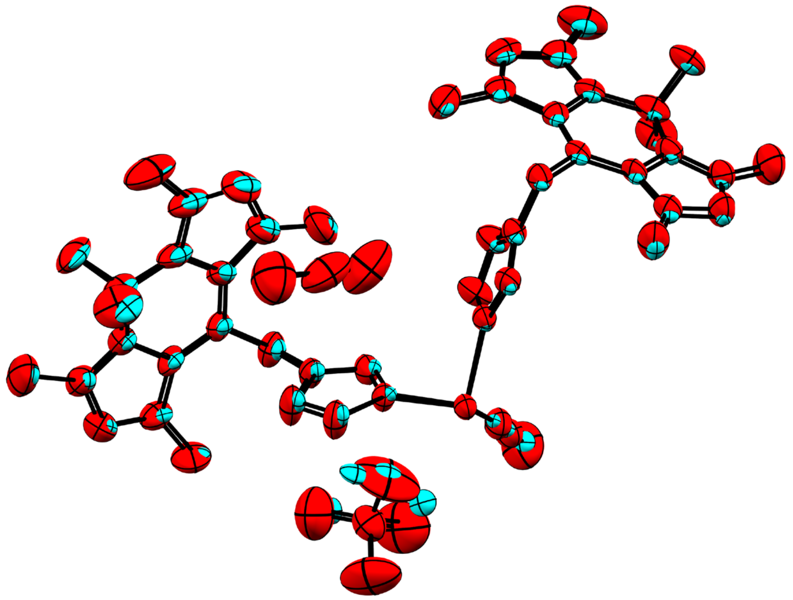

Figure S 55. Structure comparison of the asymmetric unit of coordination compound **1** at 100 K (cyan) and 300 K (red), showing differences in the disorder of the anion (ellipsoids: 50 % probability level; H-atoms are omitted for clarity).

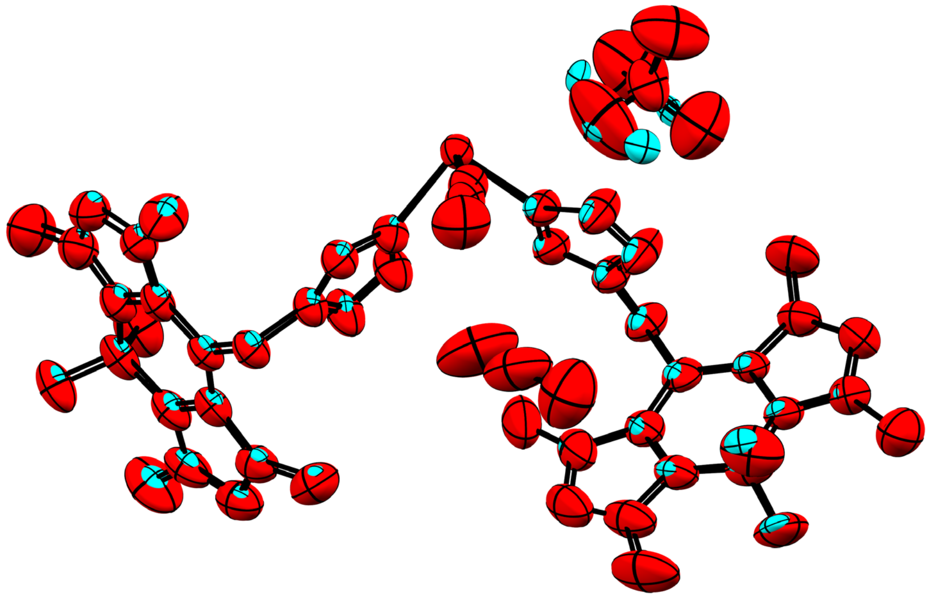

Figure S 56. Structure comparison of the asymmetric unit of coordination compound **2** at 100 K (cyan) and 300 K (red), showing differences in the disorder of the anion (ellipsoids: 50 % probability level; H-atoms are omitted for clarity).

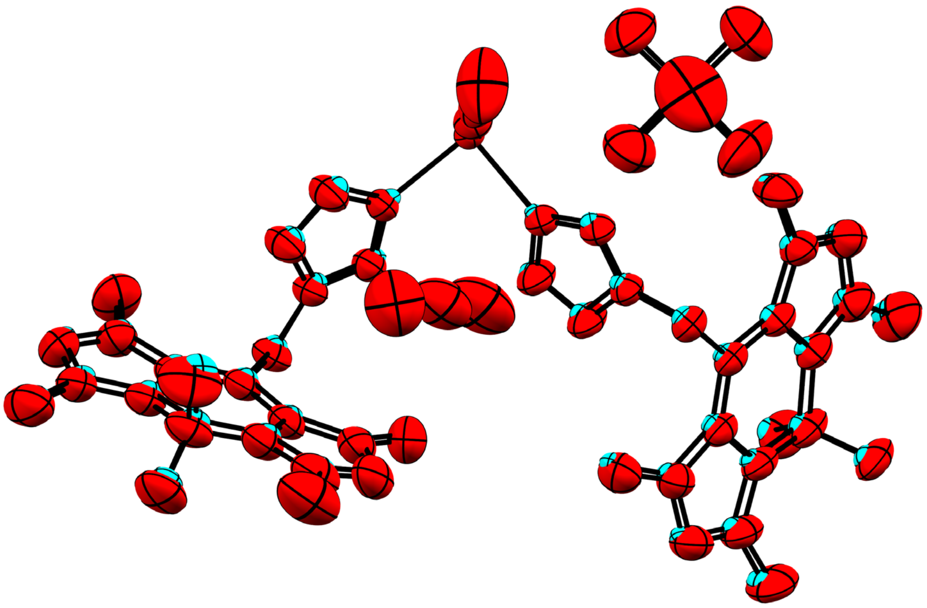

Figure S 57. Structure comparison of the asymmetric unit of coordination compound **3** at 100 K (cyan) and 300 K (red), showing hardly any differences in the spatial arrangement of the ligands, anion molecules and solvate molecules (ellipsoids: 50 % probability level; H-atoms are omitted for clarity).

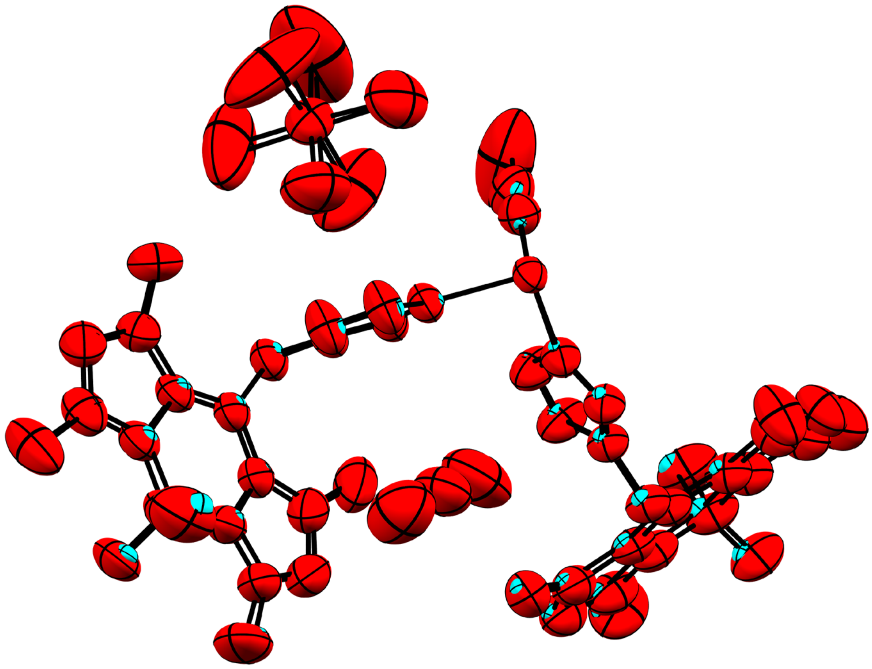

Figure S 58. Structure comparison of the asymmetric unit of coordination compound **4** at 90 K (cyan) and 300 K (red), showing hardly any differences in the spatial arrangement of the ligands, anion molecules and solvate molecules (ellipsoids: 50 % probability level; H-atoms are omitted for clarity).

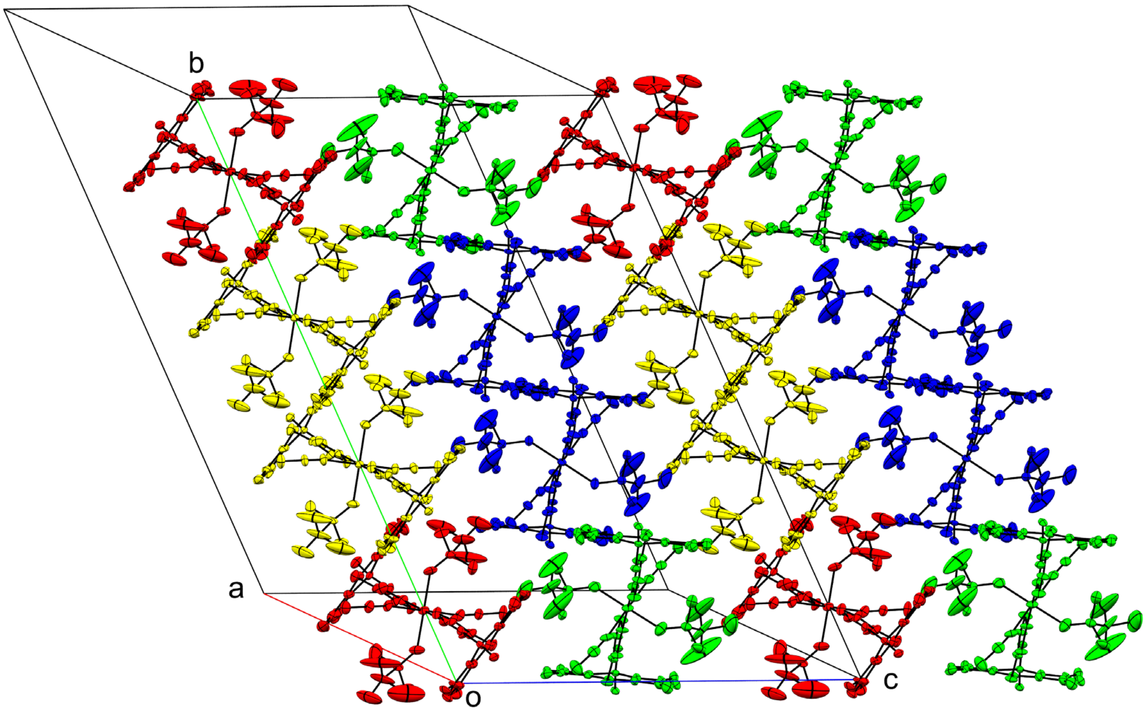

Figure S 59. Layer of coordination compound **5a** projected onto the (100) plane at 100 K (ellipsoids: 50 % probability level; color by symmetry equivalence, H-atoms are omitted for clarity).

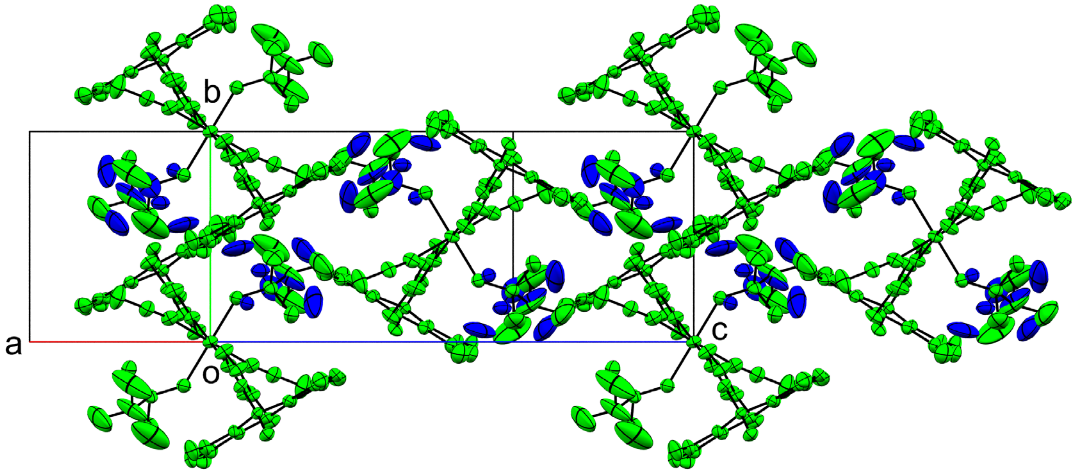

Figure S 60. Layer of coordination compound **5a** projected onto the (100) plane at 180 K (ellipsoids: 50 % probability level; color by symmetry equivalence, H-atoms are omitted for clarity)

References

(1) Huber, M.; Schöbinger, M.; Cirera, J.; Stöger, B.; Weinberger, P. Design, Synthesis and Characterization of BODIPY based 1H-Tetrazole Ligands. *European Journal of Organic Chemistry* **2025**, *28* (7). DOI: 10.1002/ejoc.202401239.

(2) Degen, T.; Sadki, M.; Bron, E.; König, U.; Nénert, G. The HighScore suite. *Powder Diffraction* **2014**, *29* (2), 13-18. DOI: 10.1017/s0885715614000840.

(3) Sheldrick, G. M. SHELXT - integrated space-group and crystal-structure determination. *Acta Crystallographica Section A: Foundations of Crystallography* **2015**, *71* (1), 3-8. DOI: 10.1107/S2053273314026370.

(4) Sheldrick, G. M. Crystal structure refinement with SHELXL. *Acta Crystallographica Section C: Crystal Structure Communications* **2015**, *71* (1), 3-8. DOI: 10.1107/S2053229614024218.

(5) Macrae, C. F.; Edgington, P. R.; McCabe, P.; Pidcock, E.; Shields, G. P.; Taylor, R.; Towler, M.; van De Streek, J. Mercury: visualization and analysis of crystal structures. *Journal of Applied Crystallography* **2006**, *39* (3), 453-457. DOI: 10.1107/S002188980600731x.

IR spectra

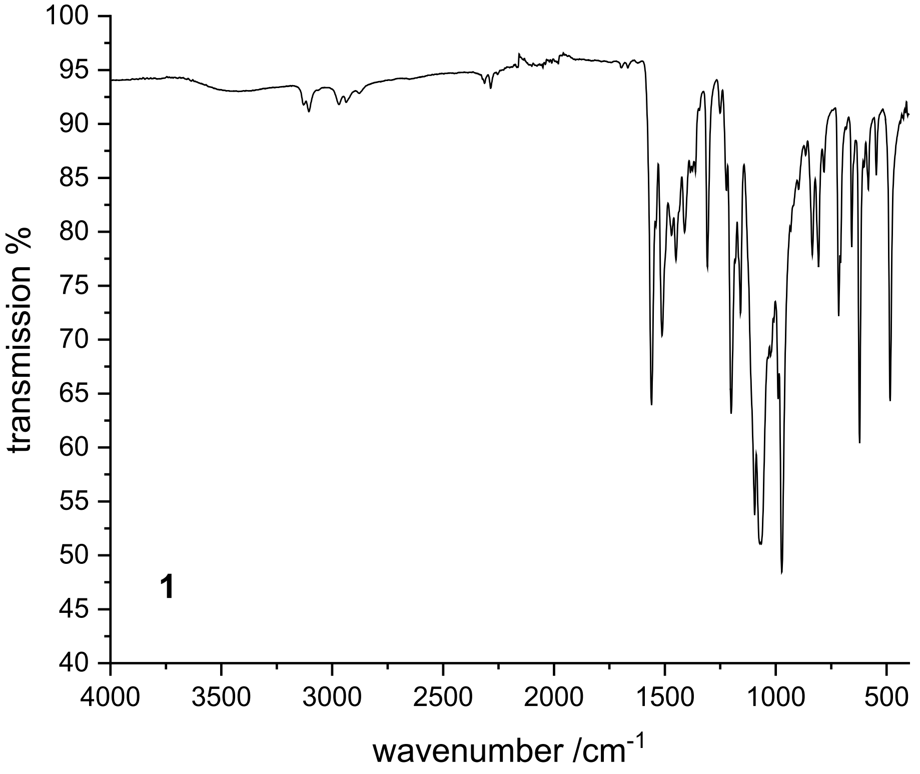

transmission %
wavenumber /cm-1
1

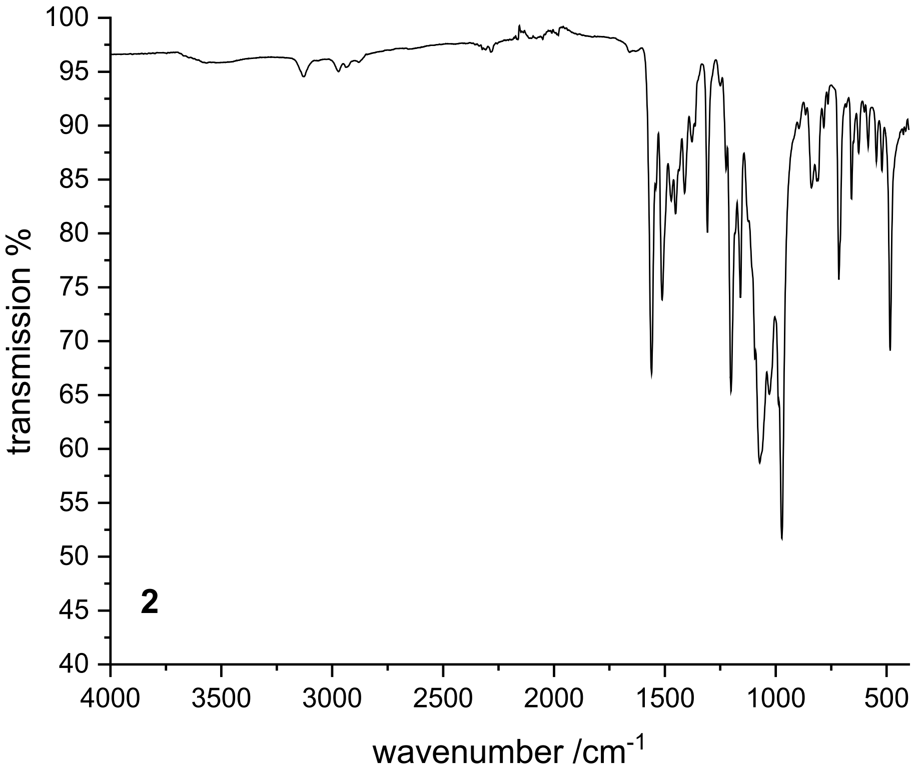

transmission %
wavenumber /cm-1
2

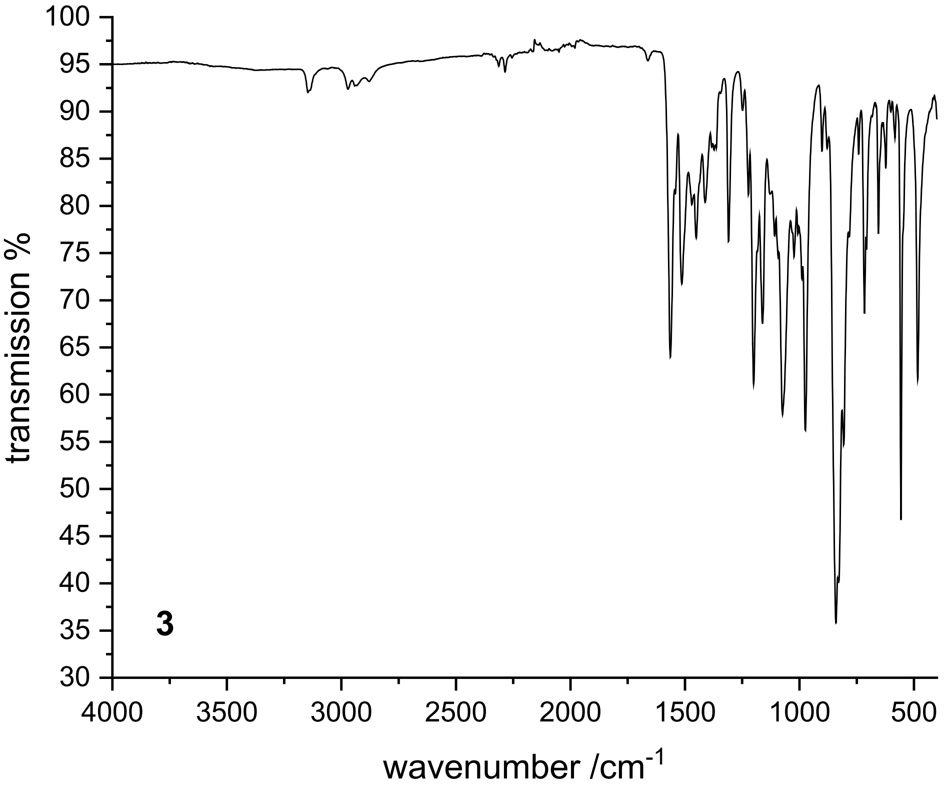

100
95
90
85
80
75
70
65
60
55
50
45
40
35
30
transmission %
4000
3500
3000
2500
2000
1500
1000
500
wavenumber /cm-1
3

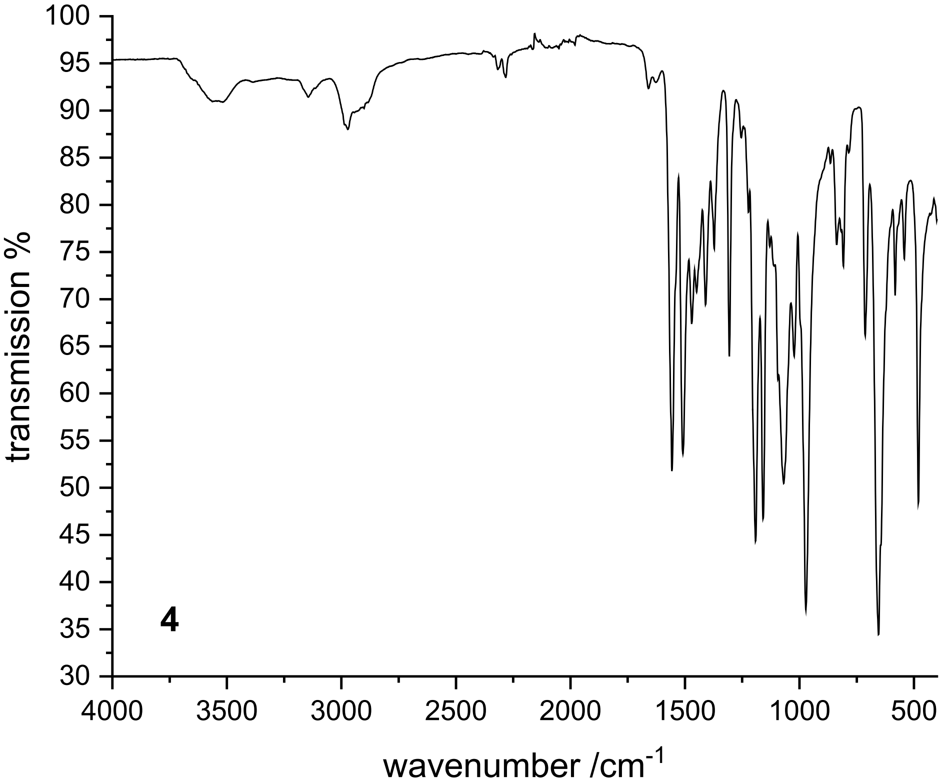

100
95
90
85
80
75
70
65
60
55
50
45
40
35
30
transmission %
4000
3500
3000
2500
2000
1500
1000
500
wavenumber /cm-1
4

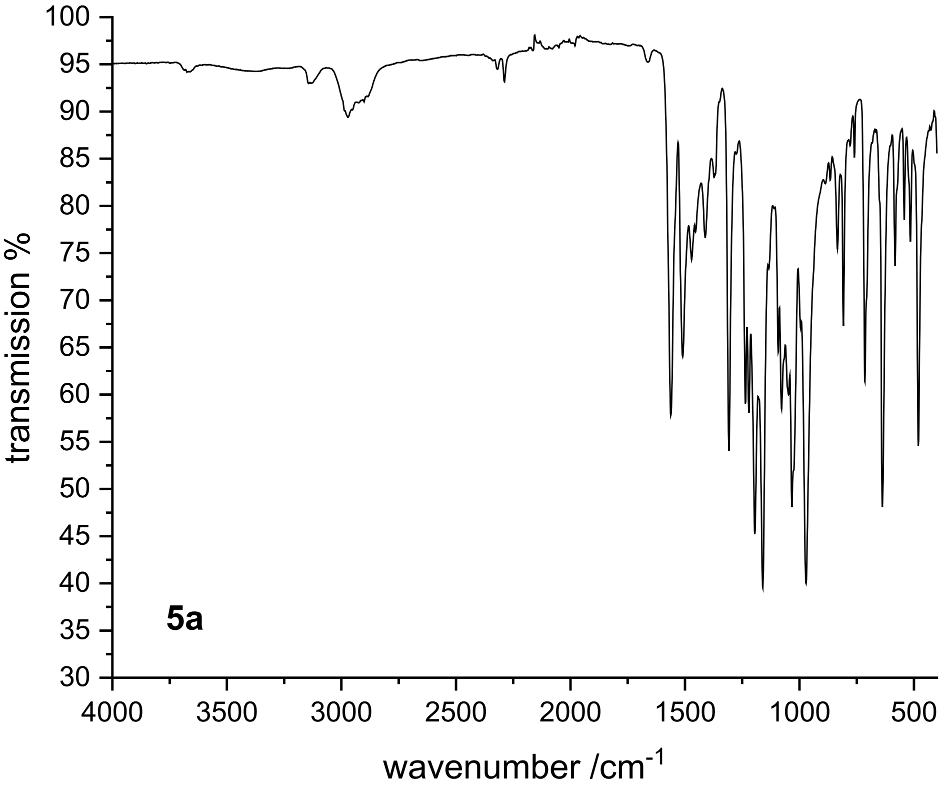

100
95
90
85
80
75
70
65
60
55
50
45
40
35
30
transmission %
5a
4000
3500
3000
2500
2000
1500
1000
500
wavenumber /cm-1

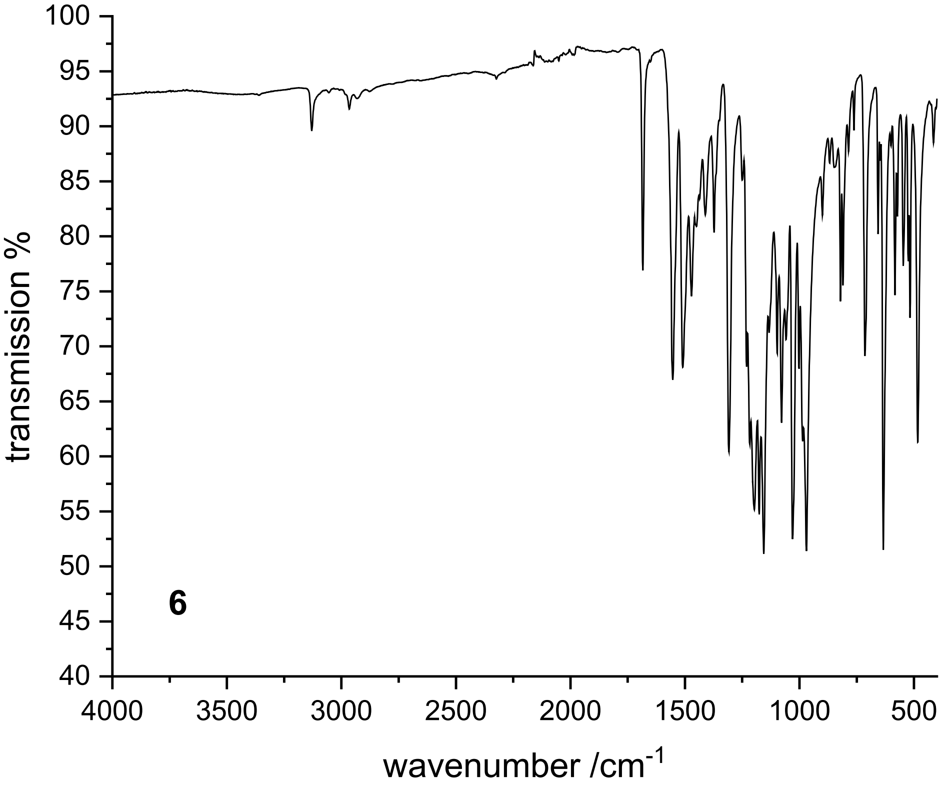

100
95
90
85
80
75
70
65
60
55
50
45
40
transmission %
6
4000
3500
3000
2500
2000
1500
1000
500
wavenumber /cm-1
